# Explorative analyses on spatial differences in the desire for social distance toward people with mental illness in a diverging city

**DOI:** 10.3389/fpubh.2023.1260118

**Published:** 2023-11-09

**Authors:** Claudia Helmert, Sven Speerforck, Toni Fleischer, Danielle Otten, Christoph Kasinger, Elmar Brähler, Holger Muehlan, Laura Altweck, Stefanie Hahm, Silke Schmidt, Matthias Reusche, Heide Glaesmer, Andreas Hinz, Nigar Reyes, Kerstin Wirkner, Christoph Engel, Georg Schomerus, Christine Ulke

**Affiliations:** ^1^Department of Psychiatry and Psychotherapy, Leipzig University, University Medical Center, Leipzig, Germany; ^2^Department of Psychosomatic Medicine and Psychotherapy, Medical Center of the Johannes Gutenberg University Mainz, Mainz, Germany; ^3^Department of Psychosomatic Medicine and Psychotherapy, Leipzig University Medical Center, Leipzig, Germany; ^4^Department of Health and Prevention, Institute of Psychology, Greifswald University, Greifswald, Germany; ^5^Institute of Medical Informatics, Statistics and Epidemiology (IMISE), Leipzig University, Leipzig, Germany; ^6^Department of Medical Psychology and Medical Sociology, Leipzig University, University Medical Center, Leipzig, Germany; ^7^Leipzig Research Centre for Civilization Diseases (LIFE), Leipzig University, Leipzig, Germany

**Keywords:** social distance, stigma, stigmatization, mental health, joint correspondence analysis, urban, public mental health

## Abstract

**Introduction:**

Stigma is an individual and societal process based on attitudes and power and relates to both spatial disparities and social distinction. In this study, we examined differences in desire for social distance toward people with mental illness within a city using social and spatial information.

**Methods:**

ANOVAs and Scheffé *post-hoc* tests analyzed varying desires for social distance toward people with mental illness within Leipzig (East Germany). Joint Correspondence Analyses (JCA) explored correspondences between desire for social distance, socio-economic status, age, life orientation, social support, duration of living in Leipzig, and shame toward having a mental illness in five city districts of Leipzig in LIFE study participants (by Leipzig Research Center for Civilization Disease, data collected 2011–2014 and 2018–2021, *n* = 521).

**Results:**

Stigma varied among Leipzig’s districts (*F*(*df* = 4) = 4.52, *p* = 0.001). JCAs showed that a higher desired social distance toward people with mental illness corresponded with spatial differences, high levels of pessimism, high shame of being mentally ill, low social support, low socio-economic status, and older age (75.74 and 81.22% explained variances).

**Conclusion:**

In terms of stigma, where people with mental illness live matters. The results identified target groups that should be addressed by appropriate intervention and prevention strategies for mental health care.

## 1. Introduction

Stigma is embedded in its cultural context and influences decisions and behavior; it shapes and is shaped by society through processes of beliefs, power, inclusion, and exclusion (1, 2). Stigma toward people with mental illness refers to “labeling, stereotyping, separation, status loss, and discrimination” (1), aggravating the consequences of mental illness and posing a barrier to mental health care (3, 4). Staiger et al. (5) investigated the double stigma of unemployment and mental illness and found that intersectionally stigmatized people reported more distress compared to singularly disadvantaged people. Else-Quest et al. (6) emphasized the importance of investigating many facets of social structures to gain information on the complex characteristics of stigma. Thinking further, intersectional approaches condense not only determinants of social inequality like gender and age but also spatial aspects, such as neighborhood, negative representations of places, and accessibility to infrastructure. These aspects additionally represent a part of health disparities and stigmatization processes (7–11). In detail, Wacquant investigates with quantitative data (for instance, from local community fact books), in-depth interviews, and ethnographic observation of territorial stigma over time (7). He points out that increasing inequalities in social determinants interrelate with spatial segregation processes and negative representation of places. People feel ashamed of living in a so-called “bad neighborhood” (for instance because of people with low socioeconomic status living there). Based on this, Halliday et al. make clear that these neighborhoods lack further in accessibility and social isolation, so they are of remarkable interest for public health research (and nevertheless under-represented in the body of research) (8). As mentioned above and the fact that intersections of stigmatized characteristics lead to stronger distress for people, it is of particular relevance to understand and overcome complex stigmatization processes.

Nevertheless, there is sparse knowledge about correspondences of spatial and social aspects and stigma toward people with mental illness. Current research seeking to close the research gap about stigma within cities provides perspectives on spatial (8) or territorial stigmatization (7, 12) as well as social dimensions of stigma. Therefore, we aim to investigate the desire for social distance toward people with mental illness in cities.

Space is shaped by people and influences people’s behavior (13). Hence, cities are realms of experience (14). Leipzig is a major city in East Germany and has areas teeming with opportunities, but it also showcases spaces marked by inequality and disadvantage (15). With more than 600,000 inhabitants in 63 city districts (16), Leipzig is one of the German cities with the fastest-growing populations (17). It is known for its art and culture scenes (18) and also for its heterogeneity (15), with the latter quality rendering Leipzig suitable for the current research question. To this end, we chose five selected city districts to portray the diversity of Leipzig’s social and cultural atmosphere: The City Center around Leipzig Central Station and the marketplace is characterized by a flow of people in shopping malls, historical buildings, and renowned concert halls; Connewitz in the South is the district with the highest proportion of forest (19) and has a flourishing independent culture scene with a history of the left-wing activist movement (20); Gohlis-North in the North of the city’s periphery has classical modern houses and a growing population (19). Grünau-North in the West of Leipzig is characterized by large-panel system buildings, and Heiterblick is an industrial area with green space.

The focus on districts as smaller units is especially important for research on the progression of social connections, distance, and networks (21). To supplement spatial data, the current study additionally investigates social features, which determine and constitute spatial varieties among individual city districts. Current analyses explore and condense past research on associations between stigma toward people with mental illness and socioeconomic status (SES) (22), social support (23), and life orientation (24), as well as associations between social distance, SES (25, 26), and social support (27). Life orientation is operationalized through pessimism regarding recovery potential in people with mental illness (28). As mentioned above, social disparities interrelate with space and mental health.

Furthermore, it is well established that cities are characterized by a higher prevalence of mental illness (29, 30) and lower stigma (31) when compared to rural areas.

Little is known about how social and spatial features correspond with stigmatization toward people with mental illness, especially within cities in Germany. We attempt to close this research gap by condensing ongoing research and adding insights into relevant features that interrelate with stigma toward people with mental illness through explorative analyses. To this end, this paper investigates characteristics associated with a desire for social distance as an expression of mental health stigma in different city districts in Leipzig.

## 2. Research questions

The current paper aims to explore possible cohesiveness and disparities in the five city districts of Leipzig mentioned above, focusing on desired social distance toward people with mental illness by combining social and spatial information on city districts. This led to the following research questions:

Are there differences in the desire for social distance toward people with mental illness between Leipzig’s city districts?

Which aspects (SES, life orientation, social support, duration of living in Leipzig, and shame of having a mental illness) constitute and correspond with the desire for social distance toward people with mental illness in different city districts of Leipzig?

## 3. Materials and methods

### 3.1. Sample

The LIFE-Adult-Study is a longitudinal cohort study by the Leipzig Research Center for Civilization Diseases (LIFE) evaluating a broad spectrum of common diseases in 10,000 randomly selected people residing in Leipzig (for further information about the LIFE-study please see (32–34)). The LIFE-study includes data on psychological and medical examinations, laboratory studies, interviews, questionnaires, and cognitive tests collected during the first wave of the study from 2011 to 2014 (32). During the second wave from 2018 to 2021 (34), new items, including items concerning the desire for social distance toward people with mental illness, were added (*n* = 2,993). Inclusion criteria were being of legal age (≥18y) and being a resident of Leipzig (32), a major city in eastern Germany with nearly 600,000 inhabitants (35). Written informed consent of all participants was obtained before data collection. The ethics committee of the Medical Faculty of Leipzig University approved the study (approval numbers 263–2009-14122009, 263/09-ff, 201/17-ek). The responsible data protection officer approved the data privacy and safety concept. (32, 34).

Urban differences were mapped to investigate inner city’s differences in attitudes and stigma (36). Leipzig has 63 city districts within nine superordinate areas. City districts as smaller, homogenous, spatial units were chosen for analyses and selected by two criteria: First, city districts had to be part of a superordinate area named after cardinal points or the city center. The second criterion was the cities with the highest number of cases. One exception is Connewitz instead of Südvorstadt for the south of Leipzig, as the participant number was nearly identical to Connewitz but not directly adjacent to the City Center. Comparing these two districts in the desire for social distance toward people with mental illness, no significant differences were found (*t*(*df* = 212) = −0.292, *p* = 0.770), justifying city districts. Finally, analyses include five of 63 city districts (*n* = 521): Leipzig’s City Center, Connewitz in the south, Gohlis-North in the north, Grünau-North in the west, and Heiterblick in the east of Leipzig.

### 3.2. Data and variables

Research data were drawn from two waves of the LIFE-adult-study (32, 34) and open-source shape files for additionally visualized maps (37).

The following measures were elicited in the first wave of the LIFE-study (2011–2014) (32): SES was operationalized according to Lampert et al. (38) through summed educational and professional status and income as social deprivation. The scale’s calculated quintiles were summarized into three categories: low, middle, and high SES (38). As life orientation is related to stigma (39, 40), dispositional and generalized pessimism and optimism were rated on a five-point Likert scale (1 “strongly disagree” to 5 “strongly agree”) as part of the Life Orientation Test (for instance “In uncertain times, I usually expect the best”) (41, adapted by 42, 43). Higher sum scores on respective instruments indicated higher levels of optimism or pessimism (44). Optimism and pessimism were seen as stable traits (41). Both scales were dichotomized at the sample’s median to depict higher and lower-than-average optimism or pessimism. Social support was operationalized by Likert-scaled answers (1 “none of the time” to 5 “all of the time”) on five items of the ENRICHD-Social Support-Instrument (ESSI) (45 adapted for a German sample by 46, 47). Analogous to Cordes et al. (47), scores were analyzed dichotomously: when two items scored less than four, participants were operationalized as lacking social support, while all other results indicated high social support. Personal master data and spatial information about the city districts the participants resided in completed the dataset.

The second LIFE survey (34) elicited the stigma variables (shame and desire for social distance) toward people with mental illness and the duration of living in Leipzig. The desire for social distance was measured using three questions that referred to acceptance regarding renting a flat to working with and living in a neighborhood with a person with mental illness, each on a five-point-Likert-scale (0 “definitely willing” to 4 “definitely unwilling,” with high values indicating a higher desired social distance) (48–50). To describe the desire for social distance, the sum scale was calculated and dichotomized using the sample’s median due to a lack of standardized reference values. Values ranged from 0 to 12, with higher scores again indicating higher social distance. An additional question investigated anticipated shame when experiencing mental illness using a Likert scale (0 “Not at all” to 4 “strongly”) (51). Shame as the emotional equivalent of self-stigma is known to be associated with the desire for social distance toward people with mental illness (52, 53). Data on the duration of each participant’s residency in Leipzig was part of the analysis, taking the known association between residential stability and the prevalence of depression into consideration (54).

We utilized Joint Correspondence Analyses (JCA) to combine social and spatial or environmental information for a multifaceted approach to stigma (55).

### 3.3. Analysis

After testing for normal distribution using the Kolmogorov–Smirnov test and homoscedasticity using the Levene test, an analysis of variance compared city district-specific mean values of desire for social distance toward people with mental illness to examine area-specific differences (56). For non-normal distributed variances, the Kruskal Wallis test compared city district-specific mean values (56). The significance level was set to 95% (α = 0.05) (56). Scheffé’s test analyzed and compared *post-hoc* contrasts (57, 58).

We created a map of reported desire for social distance toward people with mental illness in different city districts of Leipzig by combining information from the LIFE-study sample with spatial data in the City of Leipzig (37).

To explore cohering and diverging variables for these variations in desire for social distance toward people with mental illness in city districts, two JCAs were calculated (55). Ordinal and nominal data (city districts, SES, and social support) were chosen, and metric items were condensed to quartiles (referring to the sample’s distribution: age and duration of living in Leipzig) or dichotomized (referring to the sample’s median: life orientation; desire for social distance toward colleagues, neighbors, and subtenants with mental illness; and shame) (59). JCA followed a weighted least-squared algorithm with steps comparable to factor analyses for non-metric variable categories (60, 61). Data were principal-normalized as recommended for correspondence analysis with more than two variables to compare categories (62). The variable category frequencies were listed in a multiway contingency table (similar to chi-squared statistics) (63). The centroid marked the average row and column profiles (64). JCA reduces errors of diagonal values, which would depict correspondences of the same categories (55). Results were variances, inertias (λ, averaged frequencies) (55, 65), and masses (or weights, w; explaining the categories’ contributions to related variables for the whole matrix) (55, 66). By decomposing JCA’s inertia, distinct dimensions were identified and represented outlined deviations from numerical independence (64). These factors or axes were extracted; they structure the matrix of category frequencies. Explained variance for two dimensions reached more than 70%, so using more principal components was not conducive (67). For each dimension, the categories’ eigenvalues as contributions (ctr_k_%) to dimension were calculated (64).

JCAs helped to find out about characteristics corresponding with varying desired social distance toward people with mental illness and referred to five districts: City Center, Heiterblick in the east, Grünau-North in the west, Connewitz in the south, and Gohlis-North in the north of Leipzig. The first JCA included desire for social distance as a sum score and the second JCA investigated three items of the desire for social distance scale separately.

JCA results were graphically represented by a matrix that mapped the resulting dimension 1 (horizontal axis) and dimension 2 (vertical axis) (64) with data points as variable categories. The latter can be interpreted as correspondences (or distances) from the centroid (average) between each category as well as categories and axes (62, 63).

Cases with missing values were excluded from analyses as inherent in the JCA calculation procedure. Overall, there were *n* = 261 (8.72%) missing values in merged datasets on city district retrieval and *n* = 107 cases (3.58%) with missing values on the desire for social distance. We take this as a reference point to rely on van Buuren (68) to assume completely missing random data instead of imputation methods. Additionally, Diaz-Bone recommends excluding missing values in JCAs to keep analyses interpretable (59).

### 3.4. Software

All calculations were performed with Stata SE 16.0 (69) with additional packages ‘SPMAP’ to visualize spatial data (70) and ‘grc1leg’ to combine similar graphs with one legend (71).

## 4. Results

### 4.1. Sample

Of all respondents in the first wave of the LIFE-study (*n* = 10,589, 51.69% women, age: *M* = 57.61y, SD = 12.51y, Min: 18.24y, and Max: 87.83y), information on the desire for social distance was available from those additionally included in the second wave (*n* = 2,993, 51.35% women; age at the time of the second survey: *M* = 62.72y, SD = 12.97y, Min: 26.00y, and Max: 86.00y). In our sample, 15.50% (*n* = 464) reported low SES, 51.19% (*n* = 1,532) middle SES, and 22.69% (*n* = 679) high SES. The life orientation test resulted in a mean optimism score of 12.03 (SD = 2.39, Min: 3, Max: 15) and a mean pessimism score of 7.21 (SD = 2.29, Min: 3, Max: 15). ESSI score indicated low social support for 11.16% (*n* = 334) and high social support for 85.87% (*n* = 2,570) of participants. The sample included participants from 53 city districts in Leipzig who had lived there, on average, since 1988 (SD = 21.94y, Min: since 1928, Max: since 2020), while data was missing for 10 city districts.

Participants from the five districts described in the Introduction and Methods sections were included in the analysis (*n* = 521): Leipzig’s City Center with *n* = 117 participants (47.86% women; age: *M* = 61.43y), Heiterblick in the east (*n* = 91, 57.14% women; age: *M* = 64.12y), Connewitz in the south (*n* = 101, 51.49% women; age: *M*: 61.72y), Grünau-North in the west (*n* = 91, 47.86% women; age: *M* = 66.44y), and Gohlis-North in the north (*n* = 121, 53.72% women; age: *M* = 64.41y). Differences to 100% are missing values. For all descriptive information, please see Table 1.

**Table 1:** Sociodemographic characteristics for each of the five exemplary city districts of Leipzig and the whole sample, frequencies by column, and distributions (*n* = 2,993).

Variables					Leipzig’s exemplary city districts				
	Descriptives		Sample	Missings	*City Center*	*Heiterblick*	*Connewitz*	*Grünau-North*	*Gohlis-North*
*Total*		*N* (%)	2,993	261 (8.72)	117 (3.91)	91 (3.04)	101 (3.37)	91 (3.04)	121 (4.04)
Sex	Men	*N* (%)	1,407 (47.401)	113 (43.30)	61 (52.14)	39 (42.86)	49 (48.51)	46 (50.55)	56 (46.28)
	Women	*N* (%)	1,537 (51.35)	99 (37.93)	56 (47.86)	52 (57.14)	52 (51.49)	56 (47.86)	65 (53.72)
	Missings	*N* (%)	49(1.64)	49 (18.77)	0	0	0	0	0
Age		Median (Range)	64.00 (26–86)	55.50 (26–83)	61.00 (26–84)	63.00 (31–82)	60.00 (27–86)	67.00 (47–85)	66.00 (33–86)
		*M* (±SD)	62.72(±12.96)	57.18(±14.07)	61.43(±14.93)	64.12(±11.38)	61.72(±13.80)	66.44(±9.70)	64.41(±12.62)
SES^a^	Low	*N* (%)	464 (15.50)	23 (8.81)	10 (8.55)	16 (17.58)	12 (11.88)	19 (20.88)	16 (13.22)
	Middle	*N* (%)	1,532 (51.19)	109 (41.76)	49 (41.88)	60 (65.93)	56 (55.45)	51 (56.04)	74 (61.16)
	High	*N* (%)	679 (22.69)	61 (23.37)	40 (34.19)	7 (7.69)	24 (23.76)	18 (19.78)	25 (20.66)
	Missings	*N* (%)	318 (10.62)	68 (26.05)	18 (15.38)	8 (8.79)	9 (8.91)	3 (3.30)	6 (4.96)
Living in Leipzig since …		Median (Range)	1994 (1928–2020)	2003 (1941–2020)	1995 (1936–2020)	1990 (1928–2019)	1996 (1941–2019)	1987 (1940–2019)	1987 (1938–2019)
		*M* (±SD)	1987.84 (±21.94)	1996.61 (±21.40)	1989.19 (±21.18)	1987.69 (±23.56)	1986.62 (±21.81)	1986.73 (±20.08)	1986.28 (±23.75)
Optimism (LOT-Subsc)		Median (Range)	12 (3–15)	12 (6–15)	13 (3–15)	12 (3–15)	12 (5–15)	11 (3–15)	12 (3–15)
		*M* (±SD)	12.03 (±2.39)	12.11 (±2.33)	12.36 (±2.41)	11.90 (±2.25)	12.16 (±2.33)	11.19 (±2.38)	12.22 (±2.24)
	Low (3–12)	*N* (%)	1,544 (51.59)	106 (40.61)	54 (46.15)	56 (61.54)	50 (49.50)	62 (68.13)	59 (48.76)
	High (13–15)	*N* (%)	1,339 (44.74)	102 (39.08)	61 (52.14)	35 (38.46)	49 (48.51)	29 (31.87)	56 (46.28)
	Missings	*N* (%)	110 (3.68)	53 (20.31)	2 (1.71)	0	2 (1.98)	0	6 (4.96)
Pessimism (LOT Subscale)		*Median (Range)*	7 (3–15)	7 (3–14)	7 (3–14)	8 (3–13)	7 (3–13)	8 (3–11)	7 (3–13)	*M (*±*SD)*	7.21 (±2.29)	7.00 (±2.19)	6.96 (±2.23)	7.43 (±2.18)	6.95 (±2.29)	7.7 (±2.21)	7.22 (±2.20)
		Low (3–7)	*N (%)*	1,630 (54.46)	128 (49.04)	72 (61.54)	48 (52.75)	58 (57.43)	40 (43.96)	65 (53.72)
	High (8–15)	*N (%)*	1,251 (41.80)	81 (31.03)	45 (38.46)	43 (47.25)	42 (41.58)	50 (54.95)	54 (44.63)
	*Missings*	*N (%)*	112 (3.74)	52 (19.92)	0	0	1 (0.99)	1 (1.10)	2 (1.65)
	Social Support (ENRICHD-SSI)		*Median (Range)*	24 (5–25)	24 (8–25)	24 (5–25)	22 (7–25)	23 (12–25)	23 (7–25)	24 (9–25)
*M (*±SD)			22.37 (±3.41)	22.81 (±2.71)	22.67 (±3.39)	21.27 (±4.20)	22.47 (±2.70)	21.04 (±4.58)	22.63 (±3.03)
Low		*N (%)*	334 (11.16)	14 (5.36)	11 (9.40)	16 (17.58)	9 (8.91)	19 (20.88)	14 (11.57)
High		*N* (%)	2,570 (85.87)	197 (75.48)	106 (90.60)	75 (82.42)	91 (90.10)	72 (79.12)	106 (87.60)
*Missings*		*N* (%)	89 (2.93)	50 (19.16)	0	0	1 (0.99)	0	1 (0.83)
Soc. Dis. subt.		Median (Range)	3 (0–4)	3 (0–4)	3 (0–4)	4 (0–4)	3 (0–4)	4 (0–4)	3 (0–4)
		*M* (±SD)	2.96 (1.20)	2.93 (1.23)	2.84 (1.10)	3.23 (1.04)	2.97 (1.13)	3.38 (1.04)	2.85 (1.31)
Soc. Dis. coll.		Median (Range)	1 (0–4)	1 (0–4)	1 (0–4)	1 (0–4)	0 (0–4)	1 (0–4)	1 (0–4)
		*M* (±SD)	1.03 (±1.15)	1.04 (±1.17)	0.81 (±0.91)	1.09 (±1.18)	0.90 (±1.14)	1.21 (±1.19)	1 (±1.19)
*Soc. Dis. neigh.*		Median (Range)	1 (0–4)	1 (0–4)	1 (0–4)	2 (0–4)	1 (0–4)	1 (0–4)	1 (0–4)
		*M* (±SD)	1.33 (±1.20)	1.23 (±1.19)	1.26 (±1.05)	1.58 (±1.19)	1.14 (±1.21)	1.33 (±1.10)	1.25 (±1.13)
Shame		Median (Range)	1 (0–4)	1 (0–4)	1 (0–4)	1 (0–4)	1 (0–3)	1 (0–4)	1 (0–4)
		*M* (±SD)	1.22 (±1.04)	1.31 (±1.03)	1.30 (±0.98)	1.38 (±0.96)	1.09 (±1.11)	1.19 (±1.05)	1.19 (±1.05)
Soc. Dis. Sum		Median (Range)	5 (0–12)	5 (0–12)	5 (0–11)	6 (0–12)	5 (0–12)	6 (1–12)	4 (0–12)
		*M* (±SD)	5.32 (±2.84)	5.19 (±2.91)	4.89 (±2.34)	5.94 (±2.61)	5.02 (±2.85)	6.18 (±2.71)	5.10 (±2.97)

a operationalized as described in 38.

The desire for social distance varied toward subtenants (*M* = 2.96, SD = 1.20), neighbors (*M* = 1.33, SD = 1.20), and colleagues (*M* = 1.03, SD = 1.15) with mental illness. Supplementary Figures S2, S3 show city districts’ social distance toward subtenants, Supplementary Figures S4, S5 toward neighbors, and Supplementary Figures S6, S7 toward colleagues with mental illness. Comparing selected city districts resulted in varying sum scores in desire for social distance: Grünau-North (*M* = 6.18, SD = 2.71) showed the highest social distance toward people with mental illness compared to City Center (*M* = 4.89, SD = 2.34), Connewitz (*M* = 5.02, SD = 2.85), Gohlis-North (*M* = 5.10, SD = 2.97), and Heiterblick (*M* = 5.94, SD = 2.61) (ANOVA: *F*(*df* = 4) = 4.52, *p* = 0.001, Levene-Test: *F*(*df* = 4) = 1.95, *p* = 0.100). ANOVA (*F*(*df* = 4) = 3.20, *p* = 0.013, Levene-Test: *F*(*df* = 4) = 1.102, *p* = 0.355) resulted in significant variations in the desire for social distance toward neighbors with mental illness between city districts (Heiterblick: *M* = 1.58, SD = 1.19; Grünau-North: *M* = 1.61, SD = 1.19; City Center: *M* = 1.26, SD = 1.05; Gohlis-North: *M* = 1.25, SD = 1.13; Connewitz: *M* = 1.14, SD = 1.21). Desire for social distance toward subtenants with mental illness also revealed significant differences (ANOVA: *F*(*df* = 4) = 5.35, *p* = 0.002, Levene test: *F*(*df* = 4) = 4.95, *p* < 0.001, Grünau-North: *M* = 3.38, SD = 1.04, Heiterblick: *M* = 3.23, SD = 1.04, Connewitz: *M* = 2.97, SD = 1.13, Gohlis-North: *M* = 2.85, SD = 1.31, and City-Center: *M* = 2.84, SD = 1.10). *Post-hoc* tests revealed that Grünau-North, City Center, and Gohlis-North were especially important for these differences. Please see Supplementary Table S2 for detailed results. No significant differences could be reported in the desire for social distance toward colleagues with mental illness between city districts. All results are listed in Table 1. Scheffé *post-hoc* tests can be found in Supplementary Tables S1, S2.

### 4.2. Joint correspondence analyses for the desire for social distance toward people with mental illness

**Figure 1:**
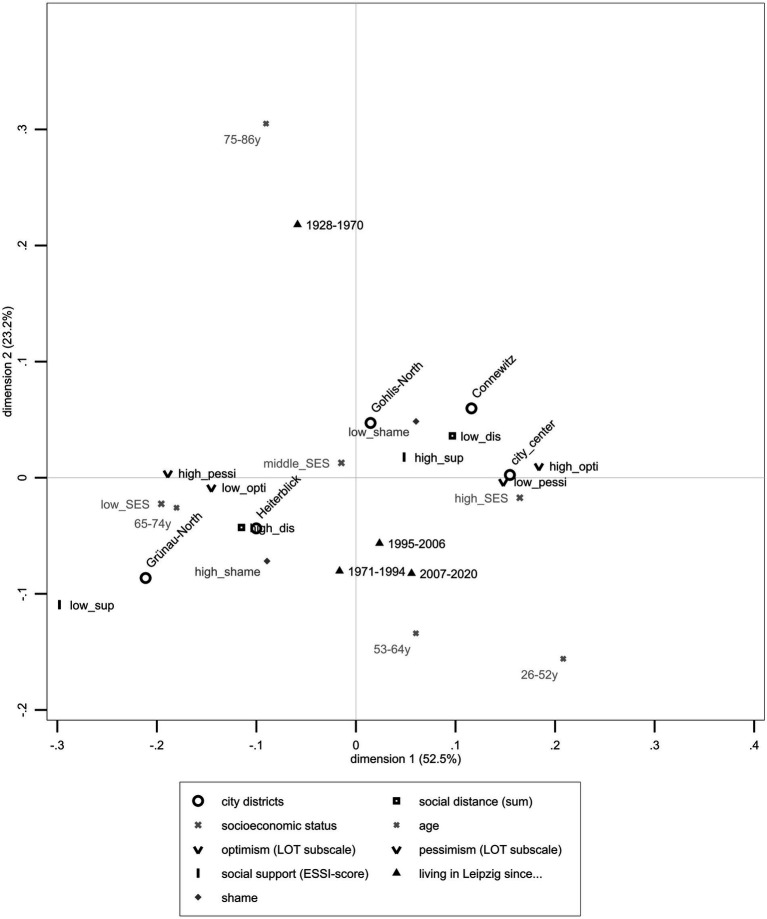
Joint Correspondence Analysis depicting sum scale on the desire for social distance toward people with mental illness, Leipzig’s exemplary districts (City center, Heiterblick, Connewitz, Gohlis-North, and Grünau-North), SES, age, life-orientation scales including dichotomized optimism and pessimism scales, dichotomized ENRICHD-Social-Support-Instrument, duration of living in Leipzig, and shame of having a mental illness based on LIFE data (*n* = 521).

As Figure 1 shows, high desire for social distance toward people with mental illness corresponded with living in Heiterblick or Grünau-North, low optimism, high pessimism, and high shame of having a mental illness. Compared to other city districts, study participants living in Grünau-North reported low social support, low SES, and high social distance toward people with mental illness. Low social distance toward people with mental illness corresponded with high social support, high optimism, low pessimism, low shame, high SES, and living in Connewitz or City Center.

Figure 1 shows JCA’s graphical results (*n* = 521) with the closest fitting of data on the first dimension (horizontal axis), which explained 52.51% (λ_1_ = 0.015) of the total variance, and the second dimension (vertical axis), which explained 23.23% (λ_2_ = 0.007) of the total variance (75.74%, λ = 0.029). For a more precise distinction, contributions to the first axis were mainly described by pessimism (ctr_%_ = 20.10%). The second dimension was based on participants’ age (ctr_%_ = 56.70%) and duration of living in Leipzig (ctr_%_ = 26.60%). Among age categories, the two extreme quartiles, oldest and youngest adults, explained most of the matrix’s variance (75–86: λ_%_ = 10.30%, 26-52y: λ_%_ = 7.40%). Supplementary Tables S3, S4 include all results concerning the first JCA with sum scales on the desire for social distance items and all variables.

**Figure 2:**
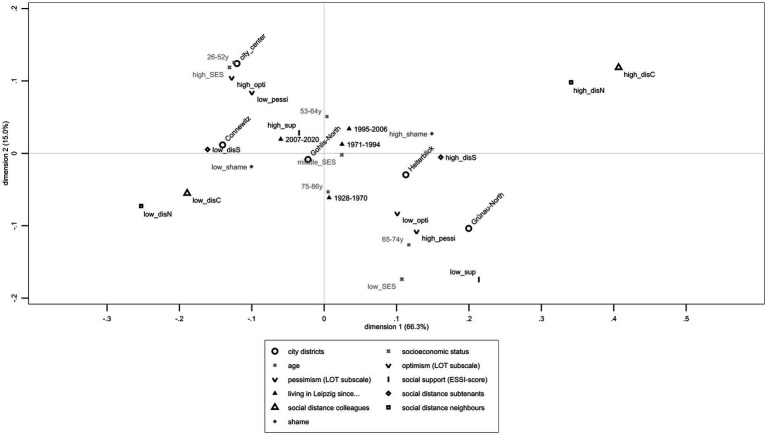
Joint Correspondence Analysis including single items on desire for social distance toward colleagues, neighbors, and subtenants with mental illness, Leipzig’s exemplary districts (City center, Heiterblick, Connewitz, Gohlis-North, and Grünau-North), SES, age, life-orientation scales including dichotomized optimism and pessimism scales, dichotomized ENRICHD-Social-Support-Instrument, duration of living in Leipzig, and shame of having a mental illness based on LIFE-data (*n* = 521).

Figure 2 shows that a high desire for social distance toward subtenants but also toward neighbors and colleagues with mental illness corresponded with a high shame of having a mental illness. Living in Heiterblick or Grünau-North, high pessimism, low optimism, low social support, and low SES as well as older age corresponded with high social distance toward subtenants with mental illness. Conversely, a low desire for social distance toward colleagues and neighbors with mental illness related to low shame, whereas a low desire for social distance toward subtenants with mental illness corresponded with high optimism, low pessimism, living in Connewitz or City Center, high SES, and high social support.

JCA explained a total variance of 81.22% through two dimensions (horizontal axis: λ_1_ = 0.024, 66.26%; vertical axis λ_2_ = 0.006, 14.96%). The desire for social distance toward neighbors (ctr_%_ = 32.20%), colleagues (ctr_%_ = 28.70%), and subtenants (ctr_%_ = 9.60%) with mental illness notably describes the horizontal axis. The second dimension can be explained by pessimism (ctr_%_ = 15.00%) and age (ctr_%_ = 14.80%). Inertias describe contributions of each variable’s categories: high desire for social distance toward colleagues (λ_%_ = 14.20%) and neighbors (λ_%_ = 13.30%) with mental illness explained most of the JCAs’ variance. Supplementary Tables S3, S5 show results on JCA with all included variables.

## 5. Discussion

Results indicate that it matters where people with mental illness live and in what socioeconomic circumstances they are embedded. We found variations in the desire for social distance toward people with mental illness corresponding to both social and spatial characteristics. The desire for social distance toward people with mental illness was lower in Leipzig’s City Center compared to other districts. Results support that there still is a stigma in cities even if urban spaces have been connoted as representing postmodern heterogeneity, diversity, and fluidity (72). Current analyses support that cities and city districts are more than spatial units: districts combine social features, which are particularly relevant when investigating social distance toward people with mental illness. Encouraged by Link and Phelan’s (1) proposal on multifaceted and multilevel approaches and Staiger et al. (5) and Else-Quest et al.’s (6) call for intersectionality in stigma research, micro (individual) and macro (urbanity-related) level factors might help understand, reflect on, and cope with stigma and desire for social distance toward people with mental illness. Investigating districts as socially constructed concepts adds insight into territorial (7, 12) and spatial stigmatization processes (8).

Because Leipzig is a growing city regarding both population and cultural diversity (15), there are still variations and progressions in and between Leipzig’s city districts (see Supplementary Figures S8–S14 in the Supplementary material for the depiction of additional characteristics of Leipzig). The five selected city districts differ not only in desire for social distance toward people with mental illness but also in SES, age, and social support implicating detailed urban and suburban research and comparisons (73). Residents in Heiterblick and Grünau-North reported low SES corresponding with high pessimism, low social support, and a high desire for social distance toward people with mental illness. These correspondences of disadvantages are supported by double stigma research (5) and by Else-Quest et al.’s (6) concept of intersectional, socially constructed categories interfering with mental health stigma. Furthermore, results condensed past findings on higher social distance toward people with mental illness to be associated with higher age (74), lower SES (22), pessimism (24), lower social support (23), and higher shame of having a mental illness (52).

Distinctions between city districts represent a self-selection bias as people choose where to live not only based on pragmatic aspects (75). Moving in different city districts as habitats might influence one’s identification with prevailing characteristics and habitus such as values and cultural diversity, as well as socioeconomic characteristics of inhabitants (76, 77). This association can be exemplarily demonstrated through Leipzig’s city district Connewitz with its long-term, leftist inhabitants (20). In the past, Connewitz was occupied by squatters who established a habitat for left-wing people (please see the election result in Supplementary Figure S13) and space for leftist discourses (78, 79). Current analyses showed high social support as well as low levels of desire for social distance toward neighbors with mental illness, accentuating a district-specific cohesion in Connewitz, regarding, for instance, shared values or lifestyles. These assumptions are consistent with past research on social segregation processes [in Leipzig: (80); but also as a postmodern phenomenon: (21)], neighborhood cohesion, and health status (81). These inner-city processes endorse interrelating social and spatial aspects as experience realms in Leipzig and other cities. Results may help establish destigmatization efforts and support people with mental illness when seeking to gain access to health care.

To conceptualize stigma, we compared a sum scale with single items of desire for social distance toward neighbors, colleagues, and subtenants with mental illness. The latter led to a more explained variance of the JCA. These results were consistent with previous research which states that items measuring the desire for social distance refer to different areas of life and that ranges of desire for social distance toward colleagues, neighbors, and subtenants cannot easily be summarized (27).

## 6. Strengths and limitations

### 6.1. Data collection

The LIFE-adult-sample was collected in two different waves. While life orientation is recognized as a stable personality trait (41), possible changes in other data, such as participants moving between city districts, could not be depicted. Due to different questionnaires and information between the two waves, longitudinal analyses and reflections were not possible. Additionally, there were dropouts over time (34).

Despite anonymized data collection, social desirability might influence participants’ response behavior to possibly objectionable questions regarding the desire for social distance toward people with mental illness. Furthermore, the desire for social distance labeled people with mental illness in general while research has shown varying desires for social distance between different disorders (26, 82), for instance, for depression and schizophrenia (27).

Sample representability is limited as participants have higher social and health status compared to recruited non-participants (33). As the sample’s health status is above average, possible results concerning mental illnesses or other health-related risk factors may be underestimated (33). Leipzig has a unique history as a city of fairs with significant influence of infrastructure and diverse perspectives from other countries (83). Additional research about past and current sociopolitical progress may help in understanding ongoing developments and problems, for instance, housing shortages because of bought-up flats or dead industries (84). Migration processes, spatial distribution, the density of schools in the city, and culturally used areas additionally reshape a district’s social structure. Leipzig currently registers remarkable demographic growth compared to other cities, especially in the East but also throughout Germany (85, 86).

### 6.2. Methodological aspects

As variables were not all distributed normally, we reported results of a non-parametrical Kruskal Wallis test. JCA allows for explorations of cross-sectional data structure and frequencies although the direction of associations or causality cannot be determined (59). Additionally, data was dichotomized and categorized, referring to the sample’s median because there was no reference data for normalization. As with all statistical calculations, correspondence analyses reduced complexity (59). The number of cases in different city districts varied; therefore, generalizations and comparative conclusions were limited (33).

## 7. Future directions

Future research should be aware of milieus or lifestyles in cities. Taking target groups into consideration, especially for anti-stigma interventions, may help to overcome social distance and support mental health literacy in marginalized groups, for instance, groups with low SES, low social support, high pessimism, and high shame toward having a mental illness.

Leipzig, with its remarkable history and current diversity, enables many possibilities for further investigations such as comparing Leipzig’s population with other urban areas. Future studies should include data over a longer period of time to gain information on fluid and stable markers of social distance and social structure in cities to detect causes and predict consequences for progressions in stigma toward people with mental illness (87, 88).

As the term ‘social distance’ refers to interpersonal and spatial information, future research should follow interdisciplinary approaches by combining historical knowledge with political, sociological, psychological, epidemiological, and geographic knowledge (89). Factors that might relate to stigma within cities are higher population densities, access to health care, or intersectional aspects (6, 90).

These approaches may help to identify target groups as well as spaces and areas that should be addressed by appropriate intervention and prevention strategies for mental health care (91, 92), like district-specific health care centers addressing spatial and social help-seeking barriers (93).

## Data availability statement

The raw data supporting the conclusions of this article will be made available by the authors, without undue reservation.

## Ethics statement

The studies involving humans were approved by the Ethics Committee of the Medical Faculty of the Leipzig University (approval numbers 263–2009-14122009, 263/09-ff, 201/17-ek). The responsible data protection officer approved data privacy and the safety concept. The studies were conducted in accordance with the local legislation and institutional requirements. The participants provided their written informed consent to participate in this study.

## Author contributions

CH: Conceptualization, Data curation, Formal analysis, Methodology, Visualization, Writing – original draft, Writing – review & editing. SS: Writing – review & editing. TF: Writing – review & editing. DO: Writing – review & editing. CK: Writing – review & editing. EB: Writing – review & editing. HM: Writing – review & editing. LA: Writing – review & editing. SH: Writing – review & editing. SS: Conceptualization, Writing – review & editing. MR: Writing – review & editing. HG: Writing – review & editing. AH: Writing – review & editing. NR: Writing – review & editing. KW: Writing – review & editing. CE: Writing – review & editing. GS: Conceptualization, Writing – review & editing. CU: Conceptualization, Data curation, Writing – review & editing.
