# Supplementary material for: Explorative analyses on spatial differences in the desire for social distance toward people with mental illness in a diverging city
Source: Front Public Health. 2023 Nov 9;11:1260118. doi: 10.3389/fpubh.2023.1260118 (PMC10665488; doi:10.3389/fpubh.2023.1260118)
Supplement: Supplementary file 1 [file Data_Sheet_1.docx]

Supplementary Material

# Supplementary Figures and Tables

## Supplementary Tables

**Supplementary Table 1**: Sociodemographic characteristics for each of the five exemplary city districts of Leipzig, frequencies by column, distributions, sample based on LIFE-data (n=2993)

|  |  |  |  | |  | | Leipzig’s city districts | | | | |
| --- | --- | --- | --- | --- | --- | --- | --- | --- | --- | --- | --- |
| Variables | Descriptives |  | Sample | Missings | | *City Center* | | *Heiterblick* | *Connewitz* | *Grünau-North* | *Gohlis-North* |
|  |  | N | 2993 | 261 | | 117 | | 91 | 101 | 91 | 121 |
|  |  | % | 100 | 8.72 | | 3.91 | | 3.04 | 3.37 | 3.04 | 4.04 |
| Sex | men | N | 1407 | 113 | | 61 | | 39 | 49 | 46 | 56 |
|  |  | % | 47.01 | 43.30 | | 52.14 | | 42.86 | 48.51 | 50.55 | 46.28 |
|  | women | N | 1537 | 99 | | 56 | | 52 | 52 | 45 | 65 |
|  |  | % | 51.35 | 37.93 | | 47.86 | | 57.14 | 51.49 | 49.45 | 53.72 |
|  | Missings | N | 49 | 49 | | 0 | | 0 | 0 | 0 | 0 |
|  |  | % | 1.64 | 18.77 | |  | |  |  |  |  |
| Age |  | Median | 64.00 | 55.50 | | 61.00 | | 63.00 | 60.00 | 67.00 | 66.00 |
|  |  | M | 62.72 | 57.18 | | 61.43 | | 64.12 | 61.72 | 66.44 | 64.41 |
|  |  | SD | 12.97 | 14.07 | | 14.93 | | 11.38 | 13.80 | 9.70 | 12.62 |
|  | Range | Min. | 26.00 | 26.00 | | 26.00 | | 31.00 | 27.00 | 47.00 | 33.00 |
|  |  | Max. | 86.00 | 83.00 | | 84.00 | | 82.00 | 86.00 | 85.00 | 86.00 |
| SES^a^ | Low | N | 464 | 23 | | 10 | | 16 | 12 | 19 | 16 |
|  |  | % | 15.50 | 8.81 | | 8.55 | | 17.58 | 11.88 | 20.88 | 13.22 |
|  | Middle | N | 1532 | 109 | | 49 | | 60 | 56 | 51 | 74 |
|  |  | % | 51.19 | 41.76 | | 41.88 | | 65.93 | 55.45 | 56.04 | 61.16 |
|  | High | N | 679 | 61 | | 40 | | 7 | 24 | 18 | 25 |
|  |  | % | 22.69 | 23.37 | | 34.19 | | 7.69 | 23.76 | 19.78 | 20.66 |
|  |  |  |  |  | |  | |  |  |  |  |
|  | Missings | N | 318 | 68 | | 18 | | 8 | 9 | 3 | 6 |
|  |  | % | 10.62 | 26.05 | | 15.38 | | 8.79 | 8.91 | 3.30 | 4.96 |
| Educational level | Still in vocational training (trainee, student) | N | 31 | 5 | | 2 | | 0 | 1 | 0 | 0 |
|  |  | % | 1.04 | 1.92 | | 1.71 | |  | 0.99 |  |  |
|  | No professional degree, no vocational training | N | 24 | 4 | | 0 | | 0 | 1 | 1 | 0 |
|  |  | % | 0.80 | 1.53 | |  | |  | 0.99 | 1.10 |  |
|  | Vocational training completed | N | 964 | 62 | | 24 | | 47 | 34 | 35 | 41 |
|  |  | % | 32.21 | 23.75 | | 20.51 | | 51.65 | 33.66 | 38.46 | 33.88 |
|  | Completed vocational school, commercial school | N | 173 | 11 | | 9 | | 6 | 6 | 2 | 7 |
|  |  | % | 5.78 | 4.21 | | 7.69 | | 6.59 | 5.94 | 2.20 | 5.79 |
|  | technical school, master school, vocational or technical academy | N | 576 | 43 | | 15 | | 12 | 18 | 25 | 22 |
|  |  | % | 19.24 | 16.38 | | 12.82 | | 13.19 | 17.82 | 27.47 | 18.18 |
|  | University of Applied Sciences degree | N | 391 | 22 | | 15 | | 14 | 12 | 8 | 15 |
|  |  | % | 13.06 | 8.43 | | 12.82 | | 15.38 | 11.88 | 8.79 | 12.40 |
|  | University degree | N | 770 | 65 | | 52 | | 11 | 28 | 20 | 36 |
|  |  | % | 25.73 | 24.90 | | 44.44 | | 12.09 | 27.72 | 21.98 | 29.75 |
|  |  |  |  |  | |  | |  |  |  |  |
|  | Other degree | N | 13 | 0 | | 0 | | 1 | 1 | 0 | 0 |
|  |  | % | 0.43 |  | |  | | 1.10 | 0.99 | 0. 0 |  |
|  | Missings | N | 51 | 49 | | 0 | | 0 | 0 | 0 | 0 |
|  |  | % | 1.70 | 18.77 | |  | |  |  |  |  |
| Living in Leipzig since … |  | Median | 1994 | 2003 | | 1995 | | 1990 | 1996 | 1987 | 1987 |
|  |  | M | 1987.84 | 1996.61 | | 1989.19 | | 1987.69 | 1986.62 | 1986.73 | 1986.28 |
|  |  | SD | 21.94 | 21.40 | | 21.18 | | 23.56 | 21.81 | 20.08 | 23.75 |
|  |  | Min. | 1928 | 1941 | | 1936 | | 1928 | 1941 | 1940 | 1938 |
|  |  | Max. | 2020 | 2020 | | 2020 | | 2019 | 2019 | 2019 | 2019 |
| Optimism (Life-Orientation-Test’s Subscale) |  | Median | 12 | 12 | | 13 | | 12 | 12 | 11 | 12 |
|  |  | M | 12.03 | 12.11 | | 12.36 | | 11.90 | 12.16 | 11.19 | 12.22 |
|  |  | SD | 2.39 | 2.33 | | 2.41 | | 2.25 | 2.33 | 2.38 | 2.24 |
|  | Range | Min. | 3 | 6 | | 3 | | 3 | 5 | 3 | 3 |
|  |  | Max. | 15 | 15 | | 15 | | 15 | 15 | 15 | 15 |
|  | Low  (und. average, 3-12) | N | 1544 | 106 | | 54 | | 56 | 50 | 62 | 59 |
|  |  | % | 51.59 | 40.61 | | 46.15 | | 61.54 | 49.50 | 68.13 | 48.76 |
|  | High  (ab. average, 13-15) | N | 1339 | 102 | | 61 | | 35 | 49 | 29 | 56 |
|  |  | % | 44.47 | 39.08 | | 52.14 | | 38.46 | 48.51 | 31.87 | 46.28 |
|  | Missings | N | 110 | 53 | | 2 | | 0 | 2 | 0 | 6 |
|  |  | % | 3.68 | 20.31 | | 1.71 | |  | 1.98 |  | 4.96 |
|  |  |  |  |  | |  | |  |  |  |  |
|  |  |  |  |  | |  | |  |  |  |  |
| Pessimism (Life-Orientation-Test’s Subscale) |  | Median | 7 | 7 | | 7 | | 8 | 7 | 8 | 7 |
|  |  | M | 7.21 | 7.00 | | 6.96 | | 7.43 | 6.95 | 7.7 | 7.22 |
|  |  | SD | 2.29 | 2.19 | | 2.23 | | 2.18 | 2.29 | 2.21 | 2.20 |
|  | Range | Min. | 3 | 3 | | 3 | | 3 | 3 | 3 | 3 |
|  |  | Max. | 15 | 14 | | 14 | | 13 | 13 | 11 | 13 |
|  | Low  (und. average, 3-7) | N | 1630 | 128 | | 72 | | 48 | 58 | 40 | 65 |
|  |  | % | 54.46 | 49.04 | | 61.54 | | 52.75 | 57.43 | 43.96 | 53.72 |
|  | high  (ab. average, 8-15) | N | 1251 | 81 | | 45 | | 43 | 42 | 50 | 54 |
|  |  | % | 41.80 | 31.03 | | 38.46 | | 47.25 | 41.58 | 54.95 | 44.63 |
|  | Missings | N | 112 | 52 | | 0 | | 0 | 1 | 1 | 2 |
|  |  | % | 3.74 | 19.92 | |  | |  | 0.99 | 1.10 | 1.65 |
| Social Support (ENRICHD-Social-Support-Instrument) |  | Median | 24 | 24 | | 24 | | 22 | 23 | 23 | 24 |
|  |  | M | 22.37 | 22.81 | | 22.67 | | 21.27 | 22.47 | 21.04 | 22.63 |
|  |  | SD | 3.41 | 2.71 | | 3.39 | | 4.20 | 2.70 | 4.58 | 3.03 |
|  | Range | Min | 5 | 8 | | 5 | | 7 | 12 | 7 | 9 |
|  |  | Max | 25 | 25 | | 25 | | 25 | 25 | 25 | 25 |
|  | Low social support | N | 334 | 14 | | 11 | | 16 | 9 | 19 | 14 |
|  |  | % | 11.16 | 5.36 | | 9.40 | | 17.58 | 8.91 | 20.88 | 11.57 |
|  | High social support | N | 2570 | 197 | | 106 | | 75 | 91 | 72 | 106 |
|  |  | % | 85.87 | 75.48 | | 90.60 | | 82.42 | 90.10 | 79.12 | 87.60 |
|  | Missings | N | 89 | 50 | | 0 | | 0 | 1 | 0 | 1 |
|  |  | % | 2.97 | 19.16 | |  | |  | 0.99 |  | 0.83 |
| Social distance towards people with mental illness: subtenant |  | Median | 3 | 3 | | 3 | | 4 | 3 | 4 | 3 |
|  |  | M | 2.96 | 2.93 | | 2.84 | | 3.23 | 2.97 | 3.38 | 2.85 |
|  |  | SD | 1.20 | 1.23 | | 1.10 | | 1.04 | 1.13 | 1.04 | 1.31 |
|  | Range | Min | 0 | 0 | | 0 | | 0 | 0 | 0 | 0 |
|  |  | Max | 4 | 4 | | 4 | | 4 | 4 | 4 | 4 |
|  | 0 “definitely willing…” | N | 122 | 14 | | 1 | | 2 | 1 | 2 | 7 |
|  |  | % | 4.08 | 5.36 | | 0.85 | | 2.20 | 0.99 | 2.20 | 5.79 |
|  | 1 | N | 258 | 21 | | 12 | | 3 | 12 | 6 | 15 |
|  |  | % | 8.62 | 8.05 | | 10.26 | | 3.30 | 11.88 | 6.59 | 12.40 |
|  | 2 | N | 655 | 55 | | 35 | | 19 | 23 | 7 | 24 |
|  |  | % | 21.88 | 21.07 | | 29.91 | | 20.88 | 22.77 | 7.69 | 19.83 |
|  | 3 | N | 458 | 45 | | 18 | | 14 | 18 | 15 | 15 |
|  |  | % | 15.30 | 17.24 | | 15.38 | | 15.38 | 17.82 | 16.48 | 12.40 |
|  | 4 “definitely unwilling…:” | N | 1430 | 120 | | 44 | | 52 | 47 | 58 | 57 |
|  |  | % | 47.78 | 45.98 | | 37.61 | | 57.14 | 46.33 | 63.74 | 47.11 |
|  | Missings | N | 70 | 6 | | 7 | | 1 | 0 | 3 | 3 |
|  |  | % | 2.34 | 2.30 | | 5.98 | | 1.10 |  | 3.30 | 2.48 |
| Social distance towards people with mental illness: *colleagues* |  | *Median* | 1 | 1 | | 1 | | 1 | 0 | 1 | 1 |
|  |  | *M* | 1.03 | 1.04 | | 0.81 | | 1.09 | 0.90 | 1.21 | 1 |
|  |  | *SD* | 1.15 | 1.17 | | 0.91 | | 1.18 | 1.14 | 1.19 | 1.19 |
|  | *Range* | *Min* | 0 | 0 | | 0 | | 0 | 0 | 0 | 0 |
|  |  | *Max* | 4 | 4 | | 4 | | 4 | 4 | 4 | 4 |
|  |  |  |  |  | |  | |  |  |  |  |
|  | 0 “definitely willing…” | *N* | 1305 | 112 | | 53 | | 40 | 51 | 35 | 57 |
|  |  | *%* | 43.60 | 42.91 | | 45.30 | | 43.96 | 50.50 | 38.46 | 47.11 |
|  | 1 | *N* | 683 | 62 | | 31 | | 15 | 24 | 14 | 25 |
|  |  | *%* | 22.82 | 23.75 | | 26.50 | | 16.48 | 23.76 | 15.38 | 20.66 |
|  | 2 | *N* | 620 | 52 | | 25 | | 24 | 16 | 27 | 23 |
|  |  | *%* | 20.72 | 19.92 | | 21.37 | | 26.37 | 15.84 | 29.67 | 19.01 |
|  | 3 | *N* | 161 | 14 | | 2 | | 6 | 5 | 7 | 8 |
|  |  | *%* | 5.38 | 5.36 | | 1.71 | | 6.59 | 4.95 | 7.69 | 6.61 |
|  | 4 “definitely unwilling…:” | *N* | 146 | 14 | | 1 | | 4 | 5 | 4 | 6 |
|  |  | *%* | 4.88 | 5.36 | | 0.85 | | 4.40 | 4.95 | 4.40 | 4.96 |
|  | *Missings* | *N* | 78 | 7 | | 5 | | 2 | 0 | 4 | 2 |
|  |  | *%* | 2.61 | 2.68 | | 4.27 | | 2.20 |  | 4.40 | 1.65 |
| Social distance towards people with mental illness: *neighbors* |  | *Median* | 1 | 1 | | 1 | | 2 | 1 | 2 | 1 |
|  |  | *M* | 1.33 | 1.23 | | 1.26 | | 1.58 | 1.14 | 1.61 | 1.25 |
|  |  | *SD* | 1.20 | 1.19 | | 1.05 | | 1.19 | 1.21 | 1.27 | 1.13 |
|  | *Range* | *Min* | 0 | 0 | | 0 | | 0 | 0 | 0 | 0 |
|  |  | *Max* | 4 | 4 | | 4 | | 4 | 4 | 4 | 4 |
|  | 0 “definitely willing…” | *N* | 911 | 87 | | 33 | | 20 | 39 | 23 | 38 |
|  |  | *%* | 30.44 | 33.33 | | 28.21 | | 21.98 | 38.61 | 25.27 | 31.40 |
|  | 1 | *N* | 786 | 71 | | 32 | | 21 | 29 | 16 | 33 |
|  |  | *%* | 26.26 | 27.20 | | 27.35 | | 23.08 | 28.71 | 17.58 | 27.27 |
|  | 2 | *N* | 784 | 63 | | 35 | | 31 | 17 | 28 | 33 |
|  |  | *%* | 26.19 | 24.14 | | 29.91 | | 34.07 | 16.83 | 30.77 | 27.27 |
|  | 3 | *N* | 225 | 12 | | 9 | | 10 | 9 | 12 | 8 |
|  |  | *%* | 7.52 | 4.60 | | 7.69 | | 10.99 | 8.91 | 13.19 | 6.61 |
|  | 4 “definitely unwilling…:” | *N* | 212 | 19 | | 3 | | 7 | 6 | 8 | 6 |
|  |  | *%* | 7.08 | 7.28 | | 2.56 | | 7.69 | 5.94 | 8.79. | 4.96 |
|  | *Missings* | *N* | 75 | 9 | | 5 | | 2 | 1 | 4 | 3 |
|  |  | *%* | 2.51 | 3.45 | | 4.27 | | 2.20 | 0.99 | 4.40 | 2.48 |
| Shame of having a mental illness |  | *Median* | 1 | 1 | | 1 | | 1 | 1 | 1 | 1 |
|  |  | *M* | 1.22 | 1.31 | | 1.30 | | 1.38 | 1.09 | 1.33 | 1.19 |
|  |  | *SD* | 1.04 | 1.03 | | 0.98 | | 0.96 | 1.11 | 1.10 | 1.05 |
|  | *Range* | *Min* | 0 | 0 | | 0 | | 0 | 0 | 0 | 0 |
|  |  | *Max* | 4 | 4 | | 4 | | 4 | 3 | 4 | 4 |
|  | 0 “not at all” | *N* | 854 | 66 | | 28 | | 16 | 40 | 25 | 38 |
|  |  | *%* | 28.53 | 25.29 | | 23.93 | | 17.58 | 39.60 | 27.47 | 31.40 |
|  | 1 | *N* | 969 | 80 | | 35 | | 36 | 29 | 25 | 34 |
|  |  | *%* | 32.38 | 30.65 | | 29.91 | | 39.56 | 28.71 | 27.47 | 28.10 |
|  | 2 | *N* | 752 | 79 | | 37 | | 25 | 15 | 24 | 33 |
|  |  | *%* | 25.13 | 30.27 | | 31.62 | | 27.47 | 14.85 | 26.37 | 27.27 |
|  | 3 | *N* | 291 | 26 | | 11 | | 11 | 17 | 12 | 11 |
|  |  | *%* | 9.72 | 9.96 | | 9.40 | | 12.09 | 16.83 | 13.19 | 9.09 |
|  | 4 “strongly” | *N* | 56 | 5 | | 1 | | 1 | 0 | 2 | 2 |
|  |  | *%* | 1.87 | 1.92 | | 0.85 | | 1.10 |  | 2.20 | 1.65 |
|  | *Missings* | *N* | 71 | 5 | | 5 | | 2 | 0 | 9 | 3 |
|  |  | *%* | 2.37 | 1.91 | | 4.27 | | 2.20 |  | 4.13 | 2.48 |
| Social Distance Sum-scale |  | *Median* | 5 | 5 | | 5 | | 6 | 5 | 6 | 4 |
|  |  | *M* | 5.32 | 5.19 | | 4.89 | | 5.94 | 5.02 | 6.18 | 5.10 |
|  |  | *SD* | 2.84 | 2.91 | | 2.34 | | 2.61 | 2.85 | 2.71 | 2.97 |
|  | *Range* | *Min* | 0 | 0 | | 0 | | 0 | 0 | 1 | 0 |
|  |  | *Max* | 12 | 12 | | 11 | | 12 | 12 | 12 | 12 |
| *Quartiles* | 0-3 | *N* | 752 | 74 | | 32 | | 13 | 35 | 10 | 38 |
|  |  | *%* | 25.13 | 28.35 | | 27.35 | | 14.29 | 34.65 | 10.99 | 31.40 |
|  | 4-5 | *N* | 798 | 66 | | 31 | | 26 | 25 | 27 | 29 |
|  |  | *%* | 26.66 | 25.29 | | 26.50 | | 28.57 | 24.75 | 29.67 | 23.97 |
|  | 6-7 | *N* | 649 | 56 | | 29 | | 25 | 18 | 17 | 21 |
|  |  | *%* | 21.68 | 21.46 | | 24.79 | | 27.47 | 17.82 | 18.68 | 17.36 |
|  | 8-12 | *N* | 687 | 56 | | 18 | | 24 | 22 | 33 | 30 |
|  |  | *%* | 22.95 | 21.56 | | 15.38 | | 26.37 | 21.78 | 36.26 | 24.79 |
|  | *Missings* | *N* | 107 | 9 | | 7 | | 3 | 1 | 4 | 3 |
|  |  | *%* | 3.58 | 3.45 | | 5.98 | | 3.30 | 0.99 | 4.40 | 2.48 |

^a^ operationalized as described in Lampert et al. 2013

**Supplementary Table 2:** Variance analyses and post-hoc-tests for social distance towards people with mental illness in each of the five exemplary city districts of Leipzig, sample based on LIFE-data (n=521)

|  | |  | | Leipzig’s city districts | | | | |
| --- | --- | --- | --- | --- | --- | --- | --- | --- |
| Variables | Row Mean- Col. Mean^b^ | | City Center | | Heiterblick | Connewitz | Grünau-North | ANOVA |
| Social distance towards people with mental illness:  subtenant | *Heiterblick* | | .397  p=0.200 | |  |  |  | F(df=4) = 5.35  p=0.002 |
|  | *Connewitz* | | .134  p=0.948 | | -.263  p=0.639 |  |  |  |
|  | *Grünau-North* | | .539  p=0.029 | | .142  p=0.953 | .404  p=0.206 |  |  |
|  | *Gohlis-North* | | 0.011  p=1.000 | | -.386  p=0.212 | -.123  p=0.959 | -.528  p=0.030 |  |
| Social distance towards people with mental illness:  colleagues | *Heiterblick* | | .277  p=0.553 | |  |  |  | F(df=4) =1.85  p=0.118 |
|  | *Connewitz* | | .088  p=0.988 | | -.189  p=0.8654 |  |  |  |
|  | *Grünau-North* | | 0.394  p=0.198 | | .117  p=0.976 | .306  p=0.483 |  |  |
|  | *Gohlis-North* | | .188  p=0.807 | | -.090  p=0.988 | .099  p=0.980 | -.207  p=0.789 |  |
| Social distance towards people with mental illness: neighbors | *Heiterblick* | | .325  p=0.425 | |  |  |  | F(df=4) =3.20  p=0.013 |
|  | *Connewitz* | | -.119  p=0.968 | | -.444  p=0.146 |  |  |  |
|  | *Grünau-North* | | .350  p=0.352 | | -.025  p=1.000 | .469  p=0.111 |  |  |
|  | *Gohlis-North* | | -.013  p=1.000 | | -.339  p=0.370 | .106  p=0.978 | -.363  p=0.302 |  |
| Social Distance Sum-scale | *Heiterblick* | | 1.052  p=0.119 | |  |  |  | F(df=4) =4.52  p=0.001 |
|  | *Connewitz* | | .130  p=0.998 | | -.923  p=0.247 |  |  |  |
|  | *Grünau-North* | | 1.293  p=0.027 | | -.241  p=0.987 | 1.164  p=0.074 |  |  |
|  | *Gohlis-North* | | .211  p=0.987 | | -.841  p=0.303 | .082  p=1.000 | -1.082  p=0.093 |  |

^b^ Scheffé-Test as post-hoc-Test for ANOVA (Scheffé 1953; Klockars & Hancock 1998)

**Supplementary Table 3**: Joint Correspondence Analyses’ axes and inertias for describing correspondences concerning desire for social distance towards people with mental illness in Leipzig’s exemplary districts (city center, Heiterblick, Connewitz, Gohlis-North, Grünau-North), SES, age, life orientation scales including dichotomized optimism and pessimism scales, dichotomized ENRICHD-Social-Support-Instrument, duration of living in Leipzig, and shame of having a mental illness, based on LIFE-data (n=521)

|  | *Joint Correspondence analysis 1^a^* | | | | |
| --- | --- | --- | --- | --- | --- |
| *Axis* | *Inertia λ_1_* | *Inertia λ_1_ %* | *Inertia λ_1_ cum.* | *Difference* | *Difference %* |
| 1 | 0.015 | 52.51 | 52.51 | 0.009 | 54.68 |
| 2 | 0.007 | 23.23 | 75.74 | 0.007 | 45.32 |
|  |  |  |  |  |  |
| ∑ | 0.029 | 100.00 |  |  |  |
|  |  |  |  |  |  |
|  | *Joint Correspondence analysis 2 ^b^* | |  |  |  |
| *Axis* | *Inertia λ_1_* | *Inertia λ_1_ %* | *Inertia λ_1_ cum.* | *Difference* | *Difference %* |
| 1 | 0.024 | 66.26 | 66.26 | 0.019 | 73.20 |
| 2 | 0.006 | 14.96 | 81.22 | 0.007 | 26.80 |
|  |  |  |  |  |  |
| ∑ | 0.037 | 100.00 |  |  |  |

^a^ including social distance towards people with mental illness as sum-scale; ^b^ with separated items on social distance towards people with mental illness as neighbors, colleagues or subtenant

**Supplementary Table 4**: Joint Correspondence Analysis with sum-scale on desire for social distance towards people with mental illness, Leipzig’s exemplary districts (city center, Heiterblick, Connewitz, Gohlis-North, Grünau- North), SES, age, life orientation scales including dichotomized optimism and pessimism scales, dichotomized ENRICHD-Social-Support-Instrument, duration of living in Leipzig, and shame of having a mental illness, contributions of categories to whole matrix (inertia), to each variable (mass), as well as coordinates and contributions to axes (ctr_%_), based on LIFE-data (n=521)

| *Categories* |  | | | | | *Axis 1* | | | | *Axis 2* | | | |
| --- | --- | --- | --- | --- | --- | --- | --- | --- | --- | --- | --- | --- | --- |
| *Labels* | *Inertia* | | | *mass* | | *x_1k_* | | *ctr_k1_%* | | *y_2k_* | | *ctr_k2_%* | |
| *City districts* |  | | |  | |  | |  | |  | |  | |
| City Center | 0.035 | | | 0.022 | | 0.155 | | 0.034 | | 0.002 | | 0.000 | |
| Heiterblick | 0.021 | | | 0.020 | | -0.100 | | 0.013 | | -0.044 | | 0.005 | |
| Connewitz | 0.020 | | | 0.022 | | 0.116 | | 0.019 | | 0.060 | | 0.012 | |
| Grünau-North | 0.049 | | | 0.021 | | -0.211 | | 0.060 | | -0.086 | | 0.023 | |
| Gohlis-North | 0.007 | | | 0.027 | | 0.015 | | 0.000 | | 0.047 | | 0.009 | |
| *Σ* | *0.132* | | | *~0.111* | |  | | *0.126* | |  | | *0.044* | |
| *Social distance sum-scale* | | | | | | | | | | | | | |
| Low (und. av., 0-5) | 0.031 | | | 0.060 | | 0.097 | | 0.037 | | 0.036 | | 0.011 | |
| High (Ab. av., 6-12) | 0.037 | | | 0.051 | | -0.115 | | 0.044 | | -0.043 | | 0.014 | |
| *Σ* | *0.068* | | | *0.111* | |  | | *0.081* | |  | | *0.025* | |
| *SES^a^* |  | | |  | |  | |  | |  | |  | |
| Low | 0.036 | | | 0.018 | | -0.196 | | 0.043 | | -0.022 | | 0.001 | |
| Middle | 0.010 | | | 0.067 | | -0.015 | | 0.001 | | 0.013 | | 0.002 | |
| High | 0.045 | | | 0.027 | | 0.165 | | 0.047 | | -0.017 | | 0.001 | |
| *Σ* | *0.091* | | | *~0.111* | |  | | *0.091* | |  | | *0.004* | |
| *Age* |  | | |  | |  | |  | |  | |  | |
| 26-52y | 0.074 | | | 0.027 | | 0.208 | | 0.075 | | -0.156 | | 0.095 | |
| 53-64y | 0.026 | | | 0.029 | | 0.060 | | 0.007 | | -0.134 | | 0.077 | |
| 65-74y | 0.046 | | | 0.026 | | -0.180 | | 0.055 | | -0.026 | | 0.003 | |
| 75-86y | 0.103 | | | 0.029 | | -0.090 | | 0.015 | | 0.305 | | 0.392 | |
| *Σ* | *0.249* | | | *~0.111* | |  | | *0.152* | |  | | *0.567* | |
| *Life-Orientation-(LOT)-Scale: Optimism* | | | | | | | | | | | | | |
| Low (und. av.,3-12) | 0.048 | | | 0.062 | | -0.145 | | 0.085 | | -0.008 | | 0.001 | |
| High (ab. av.,13-15) | 0.061 | | | 0.049 | | 0.184 | | 0.107 | | 0.010 | | 0.001 | |
| *Σ* | *0.109* | | | *0.111* | |  | | *0.192* | |  | | *0.002* | |
|  | | | | | | | | | | | | | |
| *Categories* | |  |  | | *Axis 1* | | | | *Axis 2* | | | |  |
| *Labels* | | *Inertia* | *mass* | | *x_1k_* | | *ctr_k1_%* | | *y_2k_* | | *ctr_k2_%* | |  |
| *Life-Orientation-(LOT)-Scale: Pessimism* | | | | |  | |  | |  | |  | |  |
| Low (und. av.,3-7) | | 0.053 | 0.062 | | 0.148 | | 0.088 | | -0.003 | | 0.000 | |  |
| High (ab. av.,8-12) | | 0.068 | 0.049 | | -0.189 | | 0.113 | | 0.004 | | 0.000 | |  |
| *Σ* | | *0.121* | *0.111* | |  | | *0.201* | |  | | *0.000* | |  |
| *ENRICHD social support score ESSI-Score* ^b^ | | | | | | | | | | | | |  |
| Low social support | | 0.060 | 0.016 | | -0.298 | | 0.089 | | -0.109 | | 0.027 | |  |
| High social support | | 0.010 | 0.096 | | 0.048 | | 0.014 | | 0.018 | | 0.004 | |  |
| *Σ* | | *0.070* | *~0.111* | |  | | *0.103* | |  | | *0.031* | |  |
| *Living in Leipzig since…* | |  |  | |  | |  | |  | |  | |  |
| 1928-1970 | | 0.051 | 0.028 | | -0.059 | | 0.006 | | 0.218 | | 0.197 | |  |
| 1971-1994 | | 0.017 | 0.031 | | -0.016 | | 0.001 | | -0.080 | | 0.029 | |  |
| 1995-2006 | | 0.013 | 0.023 | | 0.024 | | 0.001 | | -0.056 | | 0.011 | |  |
| 2007-2020 | | 0.023 | 0.029 | | 0.056 | | 0.006 | | -0.082 | | 0.029 | |  |
| *Σ* | | *0.104* | *0.111* | |  | | *0.014* | |  | | *0.266* | |  |
| *Shame of having a mental illness* | | | | | | | | | | | | |  |
| Low (und- average,0-1) | | 0.023 | 0.066 | | 0.060 | | 0.016 | | 0.049 | | 0.023 | |  |
| High (ab. average, 2-4) | | 0.034 | 0.045 | | -0.089 | | 0.023 | | -0.072 | | 0.034 | |  |
| *Σ* | | *0.057* | *0.111* | |  | | *0.039* | |  | | *0.057* | |  |
|  | |  |  | |  | |  | |  | |  | |  |
| *Σ Σ* | | *1* | *~1* | |  | | *~1* | |  | | *1* | |  |

^a^ operationalized as described in Lampert et al. 2013; ^b^ operationalized as described in Cordes et al. 2009

**Supplementary Table 5**: Joint Correspondence Analysis including single items on desire for social distance towards colleagues, neighbors and subtenants with mental illness, Leipzig’s exemplary districts (city center, Heiterblick, Connewitz, Gohlis-North, Grünau-North), SES, age, life orientation scales including dichotomized optimism and pessimism scales, dichotomized ENRICHD-Social-Support-Instrument, duration of living in Leipzig, and shame of having a mental illness, contributions of categories to whole matrix (inertia), to each variable (mass), as well as coordinates and contributions to axes (ctr_%_), based on LIFE-data (n=521)

| *Categories* |  | | *Axis 1* | | *Axis 2* | |
| --- | --- | --- | --- | --- | --- | --- |
| *Labels* | *Inertia%* | *mass* | *x_1k_* | *ctr_k1_%* | *y_2k_* | *ctr_k2_%* |
| *City districts* |  |  |  |  |  |  |
| City Center | 0.023 | 0.018 | -0.120 | 0.011 | 0.124 | 0.050 |
| Heiterblick | 0.013 | 0.016 | 0.113 | 0.008 | -0.030 | 0.003 |
| Connewitz | 0.014 | 0.018 | -0.140 | 0.015 | 0.012 | 0.000 |
| Grünau-North | 0.032 | 0.017 | 0.200 | 0.028 | -0.104 | 0.033 |
| Gohlis-North | 0.004 | 0.022 | -0.022 | 0.000 | -0.009 | 0.000 |
| *Σ* | *0.086* | *0.091* |  | *0.062* |  | *0.086* |
| *SES^a^* |  |  |  |  |  |  |
| Low | 0.020 | 0.014 | 0.107 | 0.007 | -0.174 | 0.079 |
| Middle | 0.006 | 0.055 | 0.024 | 0.001 | -0.002 | 0.000 |
| High | 0.029 | 0.022 | -0.131 | 0.015 | 0.119 | 0.056 |
| *Σ* | *0.055* | *0.091* |  | *0.023* |  | *0.135* |
| *Age* |  |  |  |  |  |  |
| 26-52y | 0.029 | 0.022 | -0.125 | 0.014 | 0.126 | 0.063 |
| 53-64y | 0.007 | 0.024 | 0.004 | 0.000 | 0.051 | 0.011 |
| 65-74y | 0.026 | 0.021 | 0.117 | 0.012 | -0.126 | 0.062 |
| 75-86y | 0.022 | 0.024 | 0.005 | 0.000 | -0.053 | 0.012 |
| *Σ* | *0.084* | *0.091* |  | *0.016* |  | *0.148* |
| *Life-Orientation-(LOT)-Scale: Optimism* | | | | | | |
| Low (under average, 3-12) | 0.027 | 0.051 | 0.101 | 0.021 | -0.083 | 0.063 |
| High (ab. average, 13-15) | 0.034 | 0.040 | -0.0128 | 0.027 | 0.105 | 0.080 |
| *Σ* | *0.061* | *0.091* |  | *0.048* |  | *0.143* |
| *Life-Orientation-(LOT)-Scale: Pessimism* | | |  |  |  |  |
| Low (under average, 3-7) | 0.028 | 0.051 | -0.100 | 0.021 | 0.084 | 0.066 |
| High (above average,8-12) | 0.036 | 0.040 | 0.128 | 0.027 | -0.108 | 0.084 |
| *Σ* | *0.064* | *0.091* |  | *0.048* |  | *0.150* |

|  |  |  | *Axis 1* | | *Axis 2* | |
| --- | --- | --- | --- | --- | --- | --- |
| *Labels* | *Inertia%* | *mass* | *x_1k_* | *ctr_k1_%* | *y_2k_* | *ctr_k2_%* |
| *ENRICHD social support score ESSI-Score* ^b^ | | | | | | |
| Low social support | 0.032 | 0.013 | 0.214 | 0.024 | -0.174 | 0.070 |
| High social support | 0.005 | 0.078 | -0.035 | 0.004 | 0.028 | 0.011 |
| *Σ* | *0.037* | *0.091* |  | *0.028* |  | *0.081* |
| *Living in Leipzig since…* |  |  |  |  |  |  |
| 1928-1970 | 0.021 | 0.023 | 0.007 | 0.000 | -0.061 | 0.016 |
| 1971-1994 | 0.009 | 0.025 | 0.025 | 0.001 | -0.013 | 0.001 |
| 1995-2006 | 0.010 | 0.019 | 0.034 | 0.001 | 0.034 | 0.004 |
| 2007-2020 | 0.014 | 0.024 | -0.060 | 0.004 | 0.020 | 0.002 |
| *Σ* | *0.054* | *0.091* |  | *0.006* |  | *0.023* |
| *Social distance towards people with mental illness: subtenant* | | | | | | |
| Low (under average, 0-3) | 0.036 | 0.045 | -0.161 | 0.048 | 0.005 | 0.000 |
| High (above average, 4) | 0.036 | 0.045 | 0.161 | 0.048 | -0.005 | 0.000 |
| *Σ* | *0.072* | *~0.091* |  | *0.096* |  | *0.000* |
| *Social distance towards people with mental illness: colleagues* | | | | | | |
| Low (under average, 0-1) | 0.066 | 0.062 | -0.189 | 0.091 | -0.055 | 0.034 |
| High (above average,2-4) | 0.142 | 0.029 | 0.407 | 0.196 | 0.119 | 0.074 |
| *Σ* | *0.208* | *0.091* |  | *0.287* |  | *0.108* |
| *Social distance towards people with mental illness: neighbors* | | | | | | |
| Low (under average, 0-1) | 0.099 | 0.052 | -0.253 | 0.137 | -0.073 | 0.050 |
| High (above average,2-4) | 0.133 | 0.039 | 0.341 | 0.185 | 0.098 | 0.068 |
| *Σ* | *0.232* | *0.091* |  | *0.322* |  | *0.118* |
| *Shame of having a mental illness* | | | | | | |
| Low (under average, 0-1) | 0.018 | 0.054 | -0.100 | 0.022 | -0.018 | 0.003 |
| High (above average,2-4) | 0.027 | 0.037 | 0.149 | 0.033 | 0.027 | 0.005 |
| *Σ* | *0.045* | *0.091* |  | *0.055* |  | *0.008* |
|  |  |  |  |  |  |  |
| *Σ Σ* | *~1* | *~1* |  | *~1* |  | *~1* |

^a^ operationalized as described in Lampert et al. 2013; ^b^ operationalized as described in Cordes et al. 2009

## Supplementary Figures

******Supplementary Figure 1**: Desire for social distance towards people with mental illness (quartiles) in Leipzig’s city districts with n>30 (1 City Center, 2 City Center-East, 3 City Center-Southeast, 4 City Center-South, 5 City Center-West, 6 City Center-Northwest, 7 City Center-North, 8 Schönefeld-Abtnaundorf, 9 Schönefeld-East, 10 Mockau-South, 11 Mockau-North, 12 Thekla, 13 Plaußig-Portitz, 14 Neustadt-Neuschönefeld, 15 Volksmarsdorf, 16 Anger-Crottendorf, 17 Sellerhausen-Stünz, 18 Paunsdorf, 19 Heiterblick, 20 Mölkau, 21 Engelsdorf, 22 Baalsdorf, 23 Athen-Kleinpösna, 24 Reudnitz-Thonberg, 25 Stötteritz, 26 Probstheida, 27 Meusdorf, 28 Liebertwolkwitz, 29 Holzhausen, 30 Südvorstadt, 31 Connewitz, 32 Marienbrunn, 33 Lößnig, 34 Dölitz-Dösen, 35 Schleußig, 36 Plagwitz, 37 Kleinzschocher, 38 Großzschocher, 39 Knautkleeberg-Knauthain, 40 Hartmannsdorf-Knautnaundorf, 41 Schönau, 42 Grünau-East, 43 Grünau-Mitte, 44 Grünau- Siedlung, 45 Lausen-Grünau, 46 Grünau-North, 47 Miltitz, 48 Lindenau, 49 Altlindenau, 50 Neulindenau, 51 Leutzsch, 52 Böhlitz-Ehrenberg, 53 Burghausen-Rückmarsdorf, 54 Möckern, 55 Wahren, 56 Lützschena-Stahmeln, 57 Lindenthal, 58 Gohlis-South, 59 Gohlis-Mitte, 60 Gohlis-North, 61 Eutritzsch, 62 Seehausen, 63 Wiederitzsch) (n= 2587).

Office for Statistics and Elections Leipzig (2017): Geodata of the Leipzig districts in ESRI shape format (UTM33N). Data License Germany - Attribution - Version 2.0 (www.govdata.de/dl-de/by-2-0). Leipzig. Online available at https://www.leipzig.de/fileadmin/mediendatenbank/leipzig-de/Stadt/02.1_Dez1_Allgemeine_Verwaltung/12_Statistik_und_Wahlen/Geodaten/Leipzig_Ortsteile_UTM33N.zip, last updated on 15.12.2017, last checked on 19.07.2022.

**Supplementary Figure 2:** Desire for social distance towards people with mental illness as subtenants (median) in Leipzig’s city districts (1 City Center, 2 City Center-East, 3 City Center-Southeast, 4 City Center-South, 5 City Center-West, 6 City Center-Northwest, 7 City Center-North, 8 Schönefeld-Abtnaundorf, 9 Schönefeld-East, 10 Mockau-South, 11 Mockau-North, 12 Thekla, 13 Plaußig-Portitz, 14 Neustadt-Neuschönefeld, 15 Volksmarsdorf, 16 Anger-Crottendorf, 17 Sellerhausen-Stünz, 18 Paunsdorf, 19 Heiterblick, 20 Mölkau, 21 Engelsdorf, 22 Baalsdorf, 23 Athen-Kleinpösna, 24 Reudnitz-Thonberg, 25 Stötteritz, 26 Probstheida, 27 Meusdorf, 28 Liebertwolkwitz, 29 Holzhausen, 30 Südvorstadt, 31 Connewitz, 32 Marienbrunn, 33 Lößnig, 34 Dölitz-Dösen, 35 Schleußig, 36 Plagwitz, 37 Kleinzschocher, 38 Großzschocher, 39 Knautkleeberg-Knauthain, 40 Hartmannsdorf-Knautnaundorf, 41 Schönau, 42 Grünau-East, 43 Grünau-Mitte, 44 Grünau- Siedlung, 45 Lausen-Grünau, 46 Grünau-North, 47 Miltitz, 48 Lindenau, 49 Altlindenau, 50 Neulindenau, 51 Leutzsch, 52 Böhlitz-Ehrenberg, 53 Burghausen-Rückmarsdorf, 54 Möckern, 55 Wahren, 56 Lützschena-Stahmeln, 57 Lindenthal, 58 Gohlis-South, 59 Gohlis-Mitte, 60 Gohlis-North, 61 Eutritzsch, 62 Seehausen, 63 Wiederitzsch) (n=2993).

Office for Statistics and Elections Leipzig (2017): Geodata of the Leipzig districts in ESRI shape format (UTM33N). Data License Germany - Attribution - Version 2.0 (www.govdata.de/dl-de/by-2-0). Leipzig. Online available at https://www.leipzig.de/fileadmin/mediendatenbank/leipzig-de/Stadt/02.1_Dez1_Allgemeine_Verwaltung/12_Statistik_und_Wahlen/Geodaten/Leipzig_Ortsteile_UTM33N.zip, last updated on 15.12.2017, last checked on 19.07.2022.

**Supplementary Figure 3**: Desire for social distance towards people with mental illness as subtenants (median) in Leipzig’s city districts n>30 (1 City Center, 2 City Center-East, 3 City Center-Southeast, 4 City Center-South, 5 City Center-West, 6 City Center-Northwest, 7 City Center-North, 8 Schönefeld-Abtnaundorf, 9 Schönefeld-East, 10 Mockau-South, 11 Mockau-North, 12 Thekla, 13 Plaußig-Portitz, 14 Neustadt-Neuschönefeld, 15 Volksmarsdorf, 16 Anger-Crottendorf, 17 Sellerhausen-Stünz, 18 Paunsdorf, 19 Heiterblick, 20 Mölkau, 21 Engelsdorf, 22 Baalsdorf, 23 Athen-Kleinpösna, 24 Reudnitz-Thonberg, 25 Stötteritz, 26 Probstheida, 27 Meusdorf, 28 Liebertwolkwitz, 29 Holzhausen, 30 Südvorstadt, 31 Connewitz, 32 Marienbrunn, 33 Lößnig, 34 Dölitz-Dösen, 35 Schleußig, 36 Plagwitz, 37 Kleinzschocher, 38 Großzschocher, 39 Knautkleeberg-Knauthain, 40 Hartmannsdorf-Knautnaundorf, 41 Schönau, 42 Grünau-East, 43 Grünau-Mitte, 44 Grünau- Siedlung, 45 Lausen-Grünau, 46 Grünau-North, 47 Miltitz, 48 Lindenau, 49 Altlindenau, 50 Neulindenau, 51 Leutzsch, 52 Böhlitz-Ehrenberg, 53 Burghausen-Rückmarsdorf, 54 Möckern, 55 Wahren, 56 Lützschena-Stahmeln, 57 Lindenthal, 58 Gohlis-South, 59 Gohlis-Mitte, 60 Gohlis-North, 61 Eutritzsch, 62 Seehausen, 63 Wiederitzsch) (n= 2587).

Office for Statistics and Elections Leipzig (2017): Geodata of the Leipzig districts in ESRI shape format (UTM33N). Data License Germany - Attribution - Version 2.0 (www.govdata.de/dl-de/by-2-0). Leipzig. Online available at https://www.leipzig.de/fileadmin/mediendatenbank/leipzig-de/Stadt/02.1_Dez1_Allgemeine_Verwaltung/12_Statistik_und_Wahlen/Geodaten/Leipzig_Ortsteile_UTM33N.zip, last updated on 15.12.2017, last checked on 19.07.2022.

 **Supplementary Figure 4**: Desire for social distance towards people with mental illness as neighbors (median) in Leipzig’s city districts (1 City Center, 2 City Center-East, 3 City Center-Southeast, 4 City Center-South, 5 City Center-West, 6 City Center-Northwest, 7 City Center-North, 8 Schönefeld-Abtnaundorf, 9 Schönefeld-East, 10 Mockau-South, 11 Mockau-North, 12 Thekla, 13 Plaußig-Portitz, 14 Neustadt-Neuschönefeld, 15 Volksmarsdorf, 16 Anger-Crottendorf, 17 Sellerhausen-Stünz, 18 Paunsdorf, 19 Heiterblick, 20 Mölkau, 21 Engelsdorf, 22 Baalsdorf, 23 Athen-Kleinpösna, 24 Reudnitz-Thonberg, 25 Stötteritz, 26 Probstheida, 27 Meusdorf, 28 Liebertwolkwitz, 29 Holzhausen, 30 Südvorstadt, 31 Connewitz, 32 Marienbrunn, 33 Lößnig, 34 Dölitz-Dösen, 35 Schleußig, 36 Plagwitz, 37 Kleinzschocher, 38 Großzschocher, 39 Knautkleeberg-Knauthain, 40 Hartmannsdorf-Knautnaundorf, 41 Schönau, 42 Grünau-East, 43 Grünau-Mitte, 44 Grünau- Siedlung, 45 Lausen-Grünau, 46 Grünau-North, 47 Miltitz, 48 Lindenau, 49 Altlindenau, 50 Neulindenau, 51 Leutzsch, 52 Böhlitz-Ehrenberg, 53 Burghausen-Rückmarsdorf, 54 Möckern, 55 Wahren, 56 Lützschena-Stahmeln, 57 Lindenthal, 58 Gohlis-South, 59 Gohlis-Mitte, 60 Gohlis-North, 61 Eutritzsch, 62 Seehausen, 63 Wiederitzsch) (n=2930).

Office for Statistics and Elections Leipzig (2017): Geodata of the Leipzig districts in ESRI shape format (UTM33N). Data License Germany - Attribution - Version 2.0 (www.govdata.de/dl-de/by-2-0). Leipzig. Online available at https://www.leipzig.de/fileadmin/mediendatenbank/leipzig-de/Stadt/02.1_Dez1_Allgemeine_Verwaltung/12_Statistik_und_Wahlen/Geodaten/Leipzig_Ortsteile_UTM33N.zip, last updated on 15.12.2017, last checked on 19.07.2022.

**Supplementary Figure 5**: Desire for social distance towards people with mental illness as neighbors (median) in Leipzig’s city districts n>30 (1 City Center, 2 City Center-East, 3 City Center-Southeast, 4 City Center-South, 5 City Center-West, 6 City Center-Northwest, 7 City Center-North, 8 Schönefeld-Abtnaundorf, 9 Schönefeld-East, 10 Mockau-South, 11 Mockau-North, 12 Thekla, 13 Plaußig-Portitz, 14 Neustadt-Neuschönefeld, 15 Volksmarsdorf, 16 Anger-Crottendorf, 17 Sellerhausen-Stünz, 18 Paunsdorf, 19 Heiterblick, 20 Mölkau, 21 Engelsdorf, 22 Baalsdorf, 23 Athen-Kleinpösna, 24 Reudnitz-Thonberg, 25 Stötteritz, 26 Probstheida, 27 Meusdorf, 28 Liebertwolkwitz, 29 Holzhausen, 30 Südvorstadt, 31 Connewitz, 32 Marienbrunn, 33 Lößnig, 34 Dölitz-Dösen, 35 Schleußig, 36 Plagwitz, 37 Kleinzschocher, 38 Großzschocher, 39 Knautkleeberg-Knauthain, 40 Hartmannsdorf-Knautnaundorf, 41 Schönau, 42 Grünau-East, 43 Grünau-Mitte, 44 Grünau- Siedlung, 45 Lausen-Grünau, 46 Grünau-North, 47 Miltitz, 48 Lindenau, 49 Altlindenau, 50 Neulindenau, 51 Leutzsch, 52 Böhlitz-Ehrenberg, 53 Burghausen-Rückmarsdorf, 54 Möckern, 55 Wahren, 56 Lützschena-Stahmeln, 57 Lindenthal, 58 Gohlis-South, 59 Gohlis-Mitte, 60 Gohlis-North, 61 Eutritzsch, 62 Seehausen, 63 Wiederitzsch) (n=2587).

Office for Statistics and Elections Leipzig (2017): Geodata of the Leipzig districts in ESRI shape format (UTM33N). Data License Germany - Attribution - Version 2.0 (www.govdata.de/dl-de/by-2-0). Leipzig. Online available at https://www.leipzig.de/fileadmin/mediendatenbank/leipzig-de/Stadt/02.1_Dez1_Allgemeine_Verwaltung/12_Statistik_und_Wahlen/Geodaten/Leipzig_Ortsteile_UTM33N.zip, last updated on 15.12.2017, last checked on 19.07.2022.

**Supplementary Figure 6**: Desire for social distance towards people with mental illness as colleagues (median) in Leipzig’s city districts (1 City Center, 2 City Center-East, 3 City Center-Southeast, 4 City Center-South, 5 City Center-West, 6 City Center-Northwest, 7 City Center-North, 8 Schönefeld-Abtnaundorf, 9 Schönefeld-East, 10 Mockau-South, 11 Mockau-North, 12 Thekla, 13 Plaußig-Portitz, 14 Neustadt-Neuschönefeld, 15 Volksmarsdorf, 16 Anger-Crottendorf, 17 Sellerhausen-Stünz, 18 Paunsdorf, 19 Heiterblick, 20 Mölkau, 21 Engelsdorf, 22 Baalsdorf, 23 Athen-Kleinpösna, 24 Reudnitz-Thonberg, 25 Stötteritz, 26 Probstheida, 27 Meusdorf, 28 Liebertwolkwitz, 29 Holzhausen, 30 Südvorstadt, 31 Connewitz, 32 Marienbrunn, 33 Lößnig, 34 Dölitz-Dösen, 35 Schleußig, 36 Plagwitz, 37 Kleinzschocher, 38 Großzschocher, 39 Knautkleeberg-Knauthain, 40 Hartmannsdorf-Knautnaundorf, 41 Schönau, 42 Grünau-East, 43 Grünau-Mitte, 44 Grünau- Siedlung, 45 Lausen-Grünau, 46 Grünau-North, 47 Miltitz, 48 Lindenau, 49 Altlindenau, 50 Neulindenau, 51 Leutzsch, 52 Böhlitz-Ehrenberg, 53 Burghausen-Rückmarsdorf, 54 Möckern, 55 Wahren, 56 Lützschena-Stahmeln, 57 Lindenthal, 58 Gohlis-South, 59 Gohlis-Mitte, 60 Gohlis-North, 61 Eutritzsch, 62 Seehausen, 63 Wiederitzsch) (n=2993).

Office for Statistics and Elections Leipzig (2017): Geodata of the Leipzig districts in ESRI shape format (UTM33N). Data License Germany - Attribution - Version 2.0 (www.govdata.de/dl-de/by-2-0). Leipzig. Online available at https://www.leipzig.de/fileadmin/mediendatenbank/leipzig-de/Stadt/02.1_Dez1_Allgemeine_Verwaltung/12_Statistik_und_Wahlen/Geodaten/Leipzig_Ortsteile_UTM33N.zip, last updated on 15.12.2017, last checked on 19.07.2022.

**Supplementary Figure 7**: Desire for social distance towards people with mental illness as colleagues (median) in Leipzig’s city districts n>30 (1 City Center, 2 City Center-East, 3 City Center-Southeast, 4 City Center-South, 5 City Center-West, 6 City Center-Northwest, 7 City Center-North, 8 Schönefeld-Abtnaundorf, 9 Schönefeld-East, 10 Mockau-South, 11 Mockau-North, 12 Thekla, 13 Plaußig-Portitz, 14 Neustadt-Neuschönefeld, 15 Volksmarsdorf, 16 Anger-Crottendorf, 17 Sellerhausen-Stünz, 18 Paunsdorf, 19 Heiterblick, 20 Mölkau, 21 Engelsdorf, 22 Baalsdorf, 23 Athen-Kleinpösna, 24 Reudnitz-Thonberg, 25 Stötteritz, 26 Probstheida, 27 Meusdorf, 28 Liebertwolkwitz, 29 Holzhausen, 30 Südvorstadt, 31 Connewitz, 32 Marienbrunn, 33 Lößnig, 34 Dölitz-Dösen, 35 Schleußig, 36 Plagwitz, 37 Kleinzschocher, 38 Großzschocher, 39 Knautkleeberg-Knauthain, 40 Hartmannsdorf-Knautnaundorf, 41 Schönau, 42 Grünau-East, 43 Grünau-Mitte, 44 Grünau- Siedlung, 45 Lausen-Grünau, 46 Grünau-North, 47 Miltitz, 48 Lindenau, 49 Altlindenau, 50 Neulindenau, 51 Leutzsch, 52 Böhlitz-Ehrenberg, 53 Burghausen-Rückmarsdorf, 54 Möckern, 55 Wahren, 56 Lützschena-Stahmeln, 57 Lindenthal, 58 Gohlis-South, 59 Gohlis-Mitte, 60 Gohlis-North, 61 Eutritzsch, 62 Seehausen, 63 Wiederitzsch) (n= 2587).

Office for Statistics and Elections Leipzig (2017): Geodata of the Leipzig districts in ESRI shape format (UTM33N). Data License Germany - Attribution - Version 2.0 (www.govdata.de/dl-de/by-2-0). Leipzig. Online available at https://www.leipzig.de/fileadmin/mediendatenbank/leipzig-de/Stadt/02.1_Dez1_Allgemeine_Verwaltung/12_Statistik_und_Wahlen/Geodaten/Leipzig_Ortsteile_UTM33N.zip, last updated on 15.12.2017, last checked on 19.07.2022.

**Supplementary Figure 8**: Shame of having a mental illness (median) in Leipzig’s city districts (1 City Center, 2 City Center-East, 3 City Center-Southeast, 4 City Center-South, 5 City Center-West, 6 City Center-Northwest, 7 City Center-North, 8 Schönefeld-Abtnaundorf, 9 Schönefeld-East, 10 Mockau-South, 11 Mockau-North, 12 Thekla, 13 Plaußig-Portitz, 14 Neustadt-Neuschönefeld, 15 Volksmarsdorf, 16 Anger-Crottendorf, 17 Sellerhausen-Stünz, 18 Paunsdorf, 19 Heiterblick, 20 Mölkau, 21 Engelsdorf, 22 Baalsdorf, 23 Athen-Kleinpösna, 24 Reudnitz-Thonberg, 25 Stötteritz, 26 Probstheida, 27 Meusdorf, 28 Liebertwolkwitz, 29 Holzhausen, 30 Südvorstadt, 31 Connewitz, 32 Marienbrunn, 33 Lößnig, 34 Dölitz-Dösen, 35 Schleußig, 36 Plagwitz, 37 Kleinzschocher, 38 Großzschocher, 39 Knautkleeberg-Knauthain, 40 Hartmannsdorf-Knautnaundorf, 41 Schönau, 42 Grünau-East, 43 Grünau-Mitte, 44 Grünau- Siedlung, 45 Lausen-Grünau, 46 Grünau-North, 47 Miltitz, 48 Lindenau, 49 Altlindenau, 50 Neulindenau, 51 Leutzsch, 52 Böhlitz-Ehrenberg, 53 Burghausen-Rückmarsdorf, 54 Möckern, 55 Wahren, 56 Lützschena-Stahmeln, 57 Lindenthal, 58 Gohlis-South, 59 Gohlis-Mitte, 60 Gohlis-North, 61 Eutritzsch, 62 Seehausen, 63 Wiederitzsch) (n=2993).

Office for Statistics and Elections Leipzig (2017): Geodata of the Leipzig districts in ESRI shape format (UTM33N). Data License Germany - Attribution - Version 2.0 (www.govdata.de/dl-de/by-2-0). Leipzig. Online available at https://www.leipzig.de/fileadmin/mediendatenbank/leipzig-de/Stadt/02.1_Dez1_Allgemeine_Verwaltung/12_Statistik_und_Wahlen/Geodaten/Leipzig_Ortsteile_UTM33N.zip, last updated on 15.12.2017, last checked on 19.07.2022.

**Supplementary Figure 9**: Shame of having a mental illness (median) in Leipzig’s city districts n>30 (1 City Center, 2 City Center-East, 3 City Center-Southeast, 4 City Center-South, 5 City Center-West, 6 City Center-Northwest, 7 City Center-North, 8 Schönefeld-Abtnaundorf, 9 Schönefeld-East, 10 Mockau-South, 11 Mockau-North, 12 Thekla, 13 Plaußig-Portitz, 14 Neustadt-Neuschönefeld, 15 Volksmarsdorf, 16 Anger-Crottendorf, 17 Sellerhausen-Stünz, 18 Paunsdorf, 19 Heiterblick, 20 Mölkau, 21 Engelsdorf, 22 Baalsdorf, 23 Athen-Kleinpösna, 24 Reudnitz-Thonberg, 25 Stötteritz, 26 Probstheida, 27 Meusdorf, 28 Liebertwolkwitz, 29 Holzhausen, 30 Südvorstadt, 31 Connewitz, 32 Marienbrunn, 33 Lößnig, 34 Dölitz-Dösen, 35 Schleußig, 36 Plagwitz, 37 Kleinzschocher, 38 Großzschocher, 39 Knautkleeberg-Knauthain, 40 Hartmannsdorf-Knautnaundorf, 41 Schönau, 42 Grünau-East, 43 Grünau-Mitte, 44 Grünau- Siedlung, 45 Lausen-Grünau, 46 Grünau-North, 47 Miltitz, 48 Lindenau, 49 Altlindenau, 50 Neulindenau, 51 Leutzsch, 52 Böhlitz-Ehrenberg, 53 Burghausen-Rückmarsdorf, 54 Möckern, 55 Wahren, 56 Lützschena-Stahmeln, 57 Lindenthal, 58 Gohlis-South, 59 Gohlis-Mitte, 60 Gohlis-North, 61 Eutritzsch, 62 Seehausen, 63 Wiederitzsch) (n= 2587).

Office for Statistics and Elections Leipzig (2017): Geodata of the Leipzig districts in ESRI shape format (UTM33N). Data License Germany - Attribution - Version 2.0 (www.govdata.de/dl-de/by-2-0). Leipzig. Online available at https://www.leipzig.de/fileadmin/mediendatenbank/leipzig-de/Stadt/02.1_Dez1_Allgemeine_Verwaltung/12_Statistik_und_Wahlen/Geodaten/Leipzig_Ortsteile_UTM33N.zip, last updated on 15.12.2017, last checked on 19.07.2022.

**Supplementary Figure** 10: Additional data on total offences in Leipzig‘s city districts (1 City Center, 2 City Center-East, 3 City Center-Southeast, 4 City Center-South, 5 City Center-West, 6 City Center-Northwest, 7 City Center-North, 8 Schönefeld-Abtnaundorf, 9 Schönefeld-East, 10 Mockau-South, 11 Mockau-North, 12 Thekla, 13 Plaußig-Portitz, 14 Neustadt-Neuschönefeld, 15 Volksmarsdorf, 16 Anger-Crottendorf, 17 Sellerhausen-Stünz, 18 Paunsdorf, 19 Heiterblick, 20 Mölkau, 21 Engelsdorf, 22 Baalsdorf, 23 Athen-Kleinpösna, 24 Reudnitz-Thonberg, 25 Stötteritz, 26 Probstheida, 27 Meusdorf, 28 Liebertwolkwitz, 29 Holzhausen, 30 Südvorstadt, 31 Connewitz, 32 Marienbrunn, 33 Lößnig, 34 Dölitz-Dösen, 35 Schleußig, 36 Plagwitz, 37 Kleinzschocher, 38 Großzschocher, 39 Knautkleeberg-Knauthain, 40 Hartmannsdorf-Knautnaundorf, 41 Schönau, 42 Grünau-East, 43 Grünau-Mitte, 44 Grünau- Siedlung, 45 Lausen-Grünau, 46 Grünau-North, 47 Miltitz, 48 Lindenau, 49 Altlindenau, 50 Neulindenau, 51 Leutzsch, 52 Böhlitz-Ehrenberg, 53 Burghausen-Rückmarsdorf, 54 Möckern, 55 Wahren, 56 Lützschena-Stahmeln, 57 Lindenthal, 58 Gohlis-South, 59 Gohlis-Mitte, 60 Gohlis-North, 61 Eutritzsch, 62 Seehausen, 63 Wiederitzsch), 2020 (Leipzig’s total population 31.12.2020, n= 610910, data retrieved from Office for Statistics and Elections Leipzig 2019).

Office for Statistics and Elections Leipzig (2017): Geodata of the Leipzig districts in ESRI shape format (UTM33N). Data License Germany - Attribution - Version 2.0 (www.govdata.de/dl-de/by-2-0). Leipzig. Online available at https://www.leipzig.de/fileadmin/mediendatenbank/leipzig-de/Stadt/02.1_Dez1_Allgemeine_Verwaltung/12_Statistik_und_Wahlen/Geodaten/Leipzig_Ortsteile_UTM33N.zip, last updated on 15.12.2017, last checked on 19.07.2022.

**Supplementary Figure 11**: Additional data on population size in Leipzig’s city districts (1 City Center, 2 City Center-East, 3 City Center-Southeast, 4 City Center-South, 5 City Center-West, 6 City Center-Northwest, 7 City Center-North, 8 Schönefeld-Abtnaundorf, 9 Schönefeld-East, 10 Mockau-South, 11 Mockau-North, 12 Thekla, 13 Plaußig-Portitz, 14 Neustadt-Neuschönefeld, 15 Volksmarsdorf, 16 Anger-Crottendorf, 17 Sellerhausen-Stünz, 18 Paunsdorf, 19 Heiterblick, 20 Mölkau, 21 Engelsdorf, 22 Baalsdorf, 23 Athen-Kleinpösna, 24 Reudnitz-Thonberg, 25 Stötteritz, 26 Probstheida, 27 Meusdorf, 28 Liebertwolkwitz, 29 Holzhausen, 30 Südvorstadt, 31 Connewitz, 32 Marienbrunn, 33 Lößnig, 34 Dölitz-Dösen, 35 Schleußig, 36 Plagwitz, 37 Kleinzschocher, 38 Großzschocher, 39 Knautkleeberg-Knauthain, 40 Hartmannsdorf-Knautnaundorf, 41 Schönau, 42 Grünau-East, 43 Grünau-Mitte, 44 Grünau- Siedlung, 45 Lausen-Grünau, 46 Grünau-North, 47 Miltitz, 48 Lindenau, 49 Altlindenau, 50 Neulindenau, 51 Leutzsch, 52 Böhlitz-Ehrenberg, 53 Burghausen-Rückmarsdorf, 54 Möckern, 55 Wahren, 56 Lützschena-Stahmeln, 57 Lindenthal, 58 Gohlis-South, 59 Gohlis-Mitte, 60 Gohlis-North, 61 Eutritzsch, 62 Seehausen, 63 Wiederitzsch), 2020 (Leipzig’s total population 31.12.2020, n= 610910, data retrieved from Office for Statistics and Elections Leipzig, 2019).

Office for Statistics and Elections Leipzig (2017): Geodata of the Leipzig districts in ESRI shape format (UTM33N). Data License Germany - Attribution - Version 2.0 (www.govdata.de/dl-de/by-2-0). Leipzig. Online available at https://www.leipzig.de/fileadmin/mediendatenbank/leipzig-de/Stadt/02.1_Dez1_Allgemeine_Verwaltung/12_Statistik_und_Wahlen/Geodaten/Leipzig_Ortsteile_UTM33N.zip, last updated on 15.12.2017, last checked on 19.07.2022.

**Supplementary Figure 12**: Additional data on voters’ participation in Leipzig’s city districts (1 City Center, 2 City Center-East, 3 City Center-Southeast, 4 City Center-South, 5 City Center-West, 6 City Center-Northwest, 7 City Center-North, 8 Schönefeld-Abtnaundorf, 9 Schönefeld-East, 10 Mockau-South, 11 Mockau-North, 12 Thekla, 13 Plaußig-Portitz, 14 Neustadt-Neuschönefeld, 15 Volksmarsdorf, 16 Anger-Crottendorf, 17 Sellerhausen-Stünz, 18 Paunsdorf, 19 Heiterblick, 20 Mölkau, 21 Engelsdorf, 22 Baalsdorf, 23 Athen-Kleinpösna, 24 Reudnitz-Thonberg, 25 Stötteritz, 26 Probstheida, 27 Meusdorf, 28 Liebertwolkwitz, 29 Holzhausen, 30 Südvorstadt, 31 Connewitz, 32 Marienbrunn, 33 Lößnig, 34 Dölitz-Dösen, 35 Schleußig, 36 Plagwitz, 37 Kleinzschocher, 38 Großzschocher, 39 Knautkleeberg-Knauthain, 40 Hartmannsdorf-Knautnaundorf, 41 Schönau, 42 Grünau-East, 43 Grünau-Mitte, 44 Grünau- Siedlung, 45 Lausen-Grünau, 46 Grünau-North, 47 Miltitz, 48 Lindenau, 49 Altlindenau, 50 Neulindenau, 51 Leutzsch, 52 Böhlitz-Ehrenberg, 53 Burghausen-Rückmarsdorf, 54 Möckern, 55 Wahren, 56 Lützschena-Stahmeln, 57 Lindenthal, 58 Gohlis-South, 59 Gohlis-Mitte, 60 Gohlis-North, 61 Eutritzsch, 62 Seehausen, 63 Wiederitzsch), 2019 (Leipzig’s total population 31.12.2019, n= 606 959, data retrieved from Office for Statistics and Elections Leipzig, 2019).**Supplementary Figure 13**: Additional data on election results (party with the highest percentage of votes) in Leipzig’s city districts (1 City Center, 2 City Center-East, 3 City Center-Southeast, 4 City Center-South, 5 City Center-West, 6 City Center-Northwest, 7 City Center-North, 8 Schönefeld-Abtnaundorf, 9 Schönefeld-East, 10 Mockau-South, 11 Mockau-North, 12 Thekla, 13 Plaußig-Portitz, 14 Neustadt-Neuschönefeld, 15 Volksmarsdorf, 16 Anger-Crottendorf, 17 Sellerhausen-Stünz, 18 Paunsdorf, 19 Heiterblick, 20 Mölkau, 21 Engelsdorf, 22 Baalsdorf, 23 Athen-Kleinpösna, 24 Reudnitz-Thonberg, 25 Stötteritz, 26 Probstheida, 27 Meusdorf, 28 Liebertwolkwitz, 29 Holzhausen, 30 Südvorstadt, 31 Connewitz, 32 Marienbrunn, 33 Lößnig, 34 Dölitz-Dösen, 35 Schleußig, 36 Plagwitz, 37 Kleinzschocher, 38 Großzschocher, 39 Knautkleeberg-Knauthain, 40 Hartmannsdorf-Knautnaundorf, 41 Schönau, 42 Grünau-East, 43 Grünau-Mitte, 44 Grünau- Siedlung, 45 Lausen-Grünau, 46 Grünau-North, 47 Miltitz, 48 Lindenau, 49 Altlindenau, 50 Neulindenau, 51 Leutzsch, 52 Böhlitz-Ehrenberg, 53 Burghausen-Rückmarsdorf, 54 Möckern, 55 Wahren, 56 Lützschena-Stahmeln, 57 Lindenthal, 58 Gohlis-South, 59 Gohlis-Mitte, 60 Gohlis-North, 61 Eutritzsch, 62 Seehausen, 63 Wiederitzsch), 2019 (Leipzig’s total population 31.12.2019, n= 606 959, data retrieved from Office for Statistics and Elections Leipzig, 2019).

Office for Statistics and Elections Leipzig (2017): Geodata of the Leipzig districts in ESRI shape format (UTM33N). Data License Germany - Attribution - Version 2.0 (www.govdata.de/dl-de/by-2-0). Leipzig. Online available at https://www.leipzig.de/fileadmin/mediendatenbank/leipzig-de/Stadt/02.1_Dez1_Allgemeine_Verwaltung/12_Statistik_und_Wahlen/Geodaten/Leipzig_Ortsteile_UTM33N.zip, last updated on 15.12.2017, last checked on 19.07.2022.

Office for Statistics and Elections Leipzig (2017): Geodata of the Leipzig districts in ESRI shape format (UTM33N). Data License Germany - Attribution - Version 2.0 (www.govdata.de/dl-de/by-2-0). Leipzig. Online available at https://www.leipzig.de/fileadmin/mediendatenbank/leipzig-de/Stadt/02.1_Dez1_Allgemeine_Verwaltung/12_Statistik_und_Wahlen/Geodaten/Leipzig_Ortsteile_UTM33N.zip, last updated on 15.12.2017, last checked on 19.07.2022.

**Supplementary Figure 14**: Additional data on number of schools in Leipzig’s city districts (1 City Center, 2 City Center-East, 3 City Center-Southeast, 4 City Center-South, 5 City Center-West, 6 City Center-Northwest, 7 City Center-North, 8 Schönefeld-Abtnaundorf, 9 Schönefeld-East, 10 Mockau-South, 11 Mockau-North, 12 Thekla, 13 Plaußig-Portitz, 14 Neustadt-Neuschönefeld, 15 Volksmarsdorf, 16 Anger-Crottendorf, 17 Sellerhausen-Stünz, 18 Paunsdorf, 19 Heiterblick, 20 Mölkau, 21 Engelsdorf, 22 Baalsdorf, 23 Athen-Kleinpösna, 24 Reudnitz-Thonberg, 25 Stötteritz, 26 Probstheida, 27 Meusdorf, 28 Liebertwolkwitz, 29 Holzhausen, 30 Südvorstadt, 31 Connewitz, 32 Marienbrunn, 33 Lößnig, 34 Dölitz-Dösen, 35 Schleußig, 36 Plagwitz, 37 Kleinzschocher, 38 Großzschocher, 39 Knautkleeberg-Knauthain, 40 Hartmannsdorf-Knautnaundorf, 41 Schönau, 42 Grünau-East, 43 Grünau-Mitte, 44 Grünau- Siedlung, 45 Lausen-Grünau, 46 Grünau-North, 47 Miltitz, 48 Lindenau, 49 Altlindenau, 50 Neulindenau, 51 Leutzsch, 52 Böhlitz-Ehrenberg, 53 Burghausen-Rückmarsdorf, 54 Möckern, 55 Wahren, 56 Lützschena-Stahmeln, 57 Lindenthal, 58 Gohlis-South, 59 Gohlis-Mitte, 60 Gohlis-North, 61 Eutritzsch, 62 Seehausen, 63 Wiederitzsch), 2019-2020 (Leipzig’s total population 31.12.2020, n= 610 910, data retrieved from Office for Statistics and Elections Leipzig, 2019).

Office for Statistics and Elections Leipzig (2017): Geodata of the Leipzig districts in ESRI shape format (UTM33N). Data License Germany - Attribution - Version 2.0 (www.govdata.de/dl-de/by-2-0). Leipzig. Online available at https://www.leipzig.de/fileadmin/mediendatenbank/leipzig-de/Stadt/02.1_Dez1_Allgemeine_Verwaltung/12_Statistik_und_Wahlen/Geodaten/Leipzig_Ortsteile_UTM33N.zip, last updated on 15.12.2017, last checked on 19.07.2022.
